# Network analysis, *in vivo*, and *in vitro* experiments identified the mechanisms by which *Piper longum* L. [Piperaceae] alleviates cartilage destruction, joint inflammation, and arthritic pain

**DOI:** 10.3389/fphar.2023.1282943

**Published:** 2024-01-24

**Authors:** Hee Geun Jo, Chae Yun Baek, Donghwan Kim, Sangjin Kim, Yewon Han, Chanlim Park, Ho Sueb Song, Donghun Lee

**Affiliations:** ^1^ Department of Herbal Pharmacology, College of Korean Medicine, Gachon University, Seongnam-si, Republic of Korea; ^2^ Naturalis Inc., Seongnam-si, Gyeonggi-do, Republic of Korea; ^3^ Department of Clinical Korean Medicine, Graduate School, Kyung Hee University, Seoul, Republic of Korea; ^4^ National Institute for Korean Medicine Development, Gyeongsan-si, Gyeongsangbuk-do, Republic of Korea; ^5^ Smart Software Lab Inc., Jeonju-si, Jeollabuk-do, Republic of Korea; ^6^ Department of Acupuncture and Moxibustion Medicine, College of Korean Medicine, Gachon University, Seongnam-si, Republic of Korea

**Keywords:** *Piper longum* L. [Piperaceae], network analysis, experimental verification, osteoarthritis, East Asian herbal medicine

## Abstract

Osteoarthritis (OA) is characterized by irreversible joint destruction, pain, and dysfunction. *Piper longum* L. [Piperaceae] (PL) is an East Asian herbal medicine with reported anti-inflammatory, analgesic, antioxidant, anti-stress, and anti-osteoporotic effects. This study aimed to evaluate the efficacy of PL in inhibiting pain and progressive joint destruction in OA based on its anti-inflammatory activity, and to explore its potential mechanisms using *in vivo* and *in vitro* models of OA. We predicted the potential hub targets and signaling pathways of PL through network analysis and molecular docking. Network analysis results showed that the possible hub targets of PL against OA were F2R, F3, MMP1, MMP2, MMP9, and PTGS2. The molecular docking results predicted strong binding affinities for the core compounds in PL: piperlongumine, piperlonguminine, and piperine. *In vitro* experiments showed that PL inhibited the expression of LPS-induced pro-inflammatory factors, such as F2R, F3, IL-1β, IL-6, IL-17A, MMP-1, MMP-2, MMP-3, MMP-9, MMP-13, NOS2, PTGS2, PGE2, and TNF-β. These mechanisms and effects were dose-dependent *in vivo* models. Furthermore, PL inhibited cartilage degradation in an OA-induced rat model. Thus, this study demonstrated that multiple components of PL may inhibit the multilayered pathology of OA by acting on multiple targets and pathways. These findings highlight the potential of PL as a disease-modifying OA drug candidate, which warrants further investigation.

## 1 Introduction

Osteoarthritis (OA) affects millions of people worldwide (Pigeolet et al., 2021) and is the most common type of arthritis and it is characterized by joint pain and reduced physical function (Katz et al., 2021). Recently, the economic impact of OA has increased in most nations (Al Saleh et al., 2023; Yang G. et al., 2023; Hallberg et al., 2023). According to the Global Burden of Disease study, the age-standardized incidence of OA is increasing by 0.32% annually worldwide (Jin et al., 2020). Additionally, epidemiological studies from most countries have shown that the prevalence of OA has increased by 7.8% per year between 1990 and 2019 (Yang G. et al., 2023). The inherent challenges of this disease are the relentless progression of chronic pain and gradual joint deterioration, which reduce the quality of life and ability of individuals to perform daily tasks (Mahmoudian et al., 2021; Tong et al., 2022a). Comorbid conditions associated with the chronic inflammatory response, such as cardiovascular disease, fibromyalgia, thromboembolic disease, and drug abuse due to toxicity, affect one-third of patients with OA, which increase mortality by 20%. (Katz et al., 2021; Kamps et al., 2023). As life expectancy increases, OA will become an increasingly heavy global burden, causing financial distress and patient mortality. The development of therapies that can alleviate OA symptoms and inhibit their progression is an urgent healthcare priority.

OA is traditionally recognized as a degenerative disease caused by the accumulation of mechanical stress-related damage, predominantly in musculoskeletal tissues (Wan et al., 2021). Age-related degenerative changes in the musculoskeletal system that cause pain are thought to be the dominant pathophysiological characteristics of OA (Berenbaum et al., 2018; Whittaker et al., 2021). However, recent research has shown that OA is not simply a localized anatomical lesion, but rather a disease with a complex pathophysiology centered on inflammation throughout the whole human body (Greene and Loeser, 2015). In particular, therapeutic targets that can partially reverse the course of the disease, including persistent low-grade inflammation and cartilage degeneration, have recently emerged as the most prominent pathophysiologies of OA (Mobasheri and Batt, 2016; Tong et al., 2022b). Similarly, increasing evidence suggests that low-grade intra-articular synovial inflammation directly contributes to pain and radiographic progression of OA (Sanchez-Lopez et al., 2022a). To achieve therapeutic goals, such as pain reduction and prevention of structural damage in OA, targeting anti-inflammatory activity to mitigate the pro-inflammatory mediators produced by the synovium and cartilage of affected joints holds considerable promise (Arra and Abu-Amer, 2023; Knights et al., 2023).

The current primary treatment strategy for OA is short-term symptomatic relief with acetaminophen and nonsteroidal anti-inflammatory drugs (NSAIDs) in combination with exercise (Leopoldino et al., 2019; Weng et al., 2023). However, these interventions are not only unsatisfactory in terms of effect size but they also present a substantial limitation in that they do not prevent the progression of OA, which is the most important long-term goal (Gregori et al., 2018; Mahmoudian et al., 2021). Moreover, safety concerns related to the increased risk of serious hepatic, gastrointestinal, cardiovascular, and renal side effects associated with the routine drugs represent one of the most important unmet medical needs in the treatment of patients with OA (Roberts et al., 2016; Leopoldino et al., 2019; Zádori et al., 2023). The development of disease-modifying OA drugs (DMOADs), a novel class of drugs that may potentially alleviate the disease burden and disrupt the natural history of OA, including progressive joint breakdown, is facilitated by advances in our understanding of OA pathophysiology (Oo, 2022). To date, no DMOADs have received regulatory approval for long-term efficacy and safety (Vincent, 2020). The difficulty in developing such therapies is that the inflammatory pathology of OA is not based on a single phenotype, gene target, or signaling pathway (Tong et al., 2022a; Hadzic and Beier, 2023; Yao et al., 2023). Consequently, the search for candidates that can simultaneously modulate multiple therapeutic targets and pathways involved in the pathogenesis of OA may be a prerequisite for the development of drugs that can sufficiently reduce symptoms and joint destruction, while ensuring safety during long-term administration.

Natural substances offer various pharmacologically active constituents, which have a long history of human use for the treatment of various diseases (Jo et al., 2023c; Lee et al., 2023). The unique properties of natural products, such as multitarget effects based on multiple compounds, are thought to be well adapted to the requirements of DMOADs in drug discovery (Yang et al., 2017; Luo et al., 2020; Jo et al., 2021; 2023b; Panossian, 2023; Ren et al., 2023). Especially, East Asian herbal medicines (EAHM) have a long history of use by many people in East Asia, and in recent years, considerable scientific evidence has been compiled regarding their mechanisms for improving clinical arthritis symptoms and inhibiting inflammatory pathology, which can be considered the best data source for anti-OA drug development (Li and Zhang, 2020; Wang et al., 2020; 2022; Jo et al., 2022; Liang et al., 2022; Zhang et al., 2022; Li et al., 2023). Recent EAHM studies have shown that several traditionally used herbs can inhibit the complex pathology of OA, including generalized inflammation, subchondral bone destruction, cartilage loss, and synovial defects, and this inhibition is based on their multi-component pharmacology. Among the many EAHMs, *Piper longum* L. [Piperaceae] (PL) is a promising DMOAD candidate because it has been extensively studied for its anti-inflammatory, analgesic, antioxidant, anti-stress, and anti-osteoporosis activities (Yadav et al., 2020). The oral administration of PL to rats resulted in a marked dose-dependent analgesic effect in a hot plate test (Yadav et al., 2016). Additionally, studies have shown that PL has stronger anti-inflammatory activity than indomethacin, a widely used and proven indole acid NSAID (Guo et al., 2019). Interestingly, the pharmacological activity that could contribute to the treatment of OA is thought to involve the known antioxidant activity of PL (Biswas et al., 2022). In fact, many recent studies have suggested that antioxidants may be beneficial for alleviating the symptoms and pathology of OA (Ansari et al., 2020; Tudorachi et al., 2021; Nejadhosseinian et al., 2022). However, further research is required to provide more conclusive information.

Based on the aforementioned studies, we hypothesized that PL could be a potential DMOAD candidate to inhibit both the pathological progression and symptoms of OA based on its multicomponent and multitargeted pharmacological actions. Although PL is a useful medicinal plant with a wide range of pharmacological effects that have been studied, we tested its potential as a DMOAD for the first time. To investigate this hypothesis in a multifaceted manner, we performed network analysis to comprehensively predict the pharmacological actions of PL and evaluated the effects of PL on certain biochemical parameters, inflammatory status, and morphological features of sodium iodoacetate (MIA)-induced knee OA in rats. Additionally, we evaluated the analgesic effects in an acetic acid-induced mouse writhing model and induced the pathophysiology of OA with *in vitro* experiments using various inflammatory cytokines and catabolic markers to predict and verify possible mechanisms of action.

## 2 Materials and methods

### 2.1 Network analysis prediction of PL for OA

#### 2.1.1 Screening of active compounds of PL against OA

The potential active compounds of PL were searched for in five databases: the Traditional Chinese Medicine Systems Pharmacology Database and Analysis platform (TCMSP, https://old.tcmsp-e.com/tcmsp.php), Traditional Chinese Medicine Information database (TCM-ID, http://bidd.group/TCMID/), Encyclopedia of Traditional Chinese Medicine (ETCM, http://www.tcmip.cn/ETCM/), Linking of Traditional Chinese Medicine with Modern Medicine at the Molecular and Phenotypic Levels (LTM-TCM, http://cloud.tasly.com/#/tcm/home), and High-Throughput Experiment and Reference-Guided Database of Traditional Chinese Medicine (HERB, http://herb.ac.cn/) (Xue et al., 2013; Ru et al., 2014; Xu et al., 2019; Fang et al., 2021; Li et al., 2022). The compounds collected from each database were standardized using PubChem (https://pubchem.ncbi.nlm.nih.gov/) and duplicates were removed. Subsequently, a drug-like (DL) threshold of 0.18 and an oral bioavailability (OB) threshold of 30% were used to screen candidate active compounds for analysis (Xu et al., 2012; Gu et al., 2020).

#### 2.1.2 Common target prediction of PL against OA

Swiss TargetPrediction (http://www.swisstargetprediction.ch) was used to compile a list of potential PL targets (Daina et al., 2019). Using the term “osteoarthritis” and “OA”, information on OA-related target genes was retrieved from DrugBank (https://www.drugbank.ca/), GeneCards (http://www.genecards.org), OMIM (https://omim.org/), and TTD (https://db.idrblab.org/ttd/) (Zhou et al., 2022). Only targets in GeneCards with a score ≥10 were screened (Stelzer et al., 2016). The “*Homo sapiens*” species filter in the Uniprot database (http://www.uniprot.org) was used to standardize all potential target information (The UniProt Consortium, 2021). Venn diagrams of common targets between PL and OA were generated using the Bioinformatics and Evolutionary Genomics website (https://bioinformatics.psb.ugent.be/webtools/Venn/).

#### 2.1.3 Protein–protein interaction (PPI) network construction

For the identified common targets, a PPI network was generated using the String database (version 11.5; https://string-db.org/), with the minimum required interaction score is set to 0.4 (medium confidence). For topological analysis of the PPI network, the PPI network was acquired, irrelevant protein nodes were removed, and the data were imported into Cytoscape (version 3.9.1) and Cytohubba plug-ins (Shannon et al., 2003; Chin et al., 2014). Genes with the top 25% degree of centrality were selected as hub targets.

#### 2.1.4 Gene ontology (GO) and kyoto encyclopedia of genes and genomes (KEGG) analysis

Metascape (https://metascape.org/gp/index.html) incorporates more than 40 gene functional annotation databases into a web-based tool for gene enrichment analysis (Zhou et al., 2019). Gene Ontology (GO) and Kyoto Encyclopedia of Genes and Genomes (KEGG) analyses were performed using this tool. After limiting the species to “*H. sapiens*,” setting the cutoff *p*-value at 0.01 and the minimum overlap at three for enrichment analysis, encompassing biological processe (BP), cellular component (CC), molecular function (MF), and KEGG pathways, we investigated the gene symbols of common targets in Metascape. The results were visualized using the Science and Research Plot platform (SRPLOT, http://www.bioinformatics.com.cn/en?*p*=6) and the KEGG mapper (https://www.genome.jp/kegg/mapper/) was used to explore the underlying molecular mechanisms (Kanehisa et al., 2022).

#### 2.1.5 Establishment of a drug-compound-target-pathway-disease (D-C-T-P-D) network

Cytoscape was used to generate the D-C-T-P-D network model, and information on drugs, compounds, genes, pathways, and diseases was used as appropriate. We inserted the enriched key pathways to establish the relationships between these elements and used degree values to complete the regulatory network.

#### 2.1.6 Molecular docking

SwissDock was used for the molecular docking prediction analysis of the key compounds in PL and key targets in OA (Grosdidier et al., 2011). The chemical structures of the key compounds were downloaded from the PubChem database in SDF 3D format and converted to the mol2 format using OpenBabel software for analysis. Target protein resolution and release time data were obtained from the RCSB Protein Data Bank (RCSB.org) (Berman et al., 2000; Burley et al., 2023). Specific compound-target binding sites and atomic distances were expressed using the UCSF chimera software (Pettersen et al., 2004).

### 2.2 PL extract (PLE) preparation

Dried PL fruit (CK20-G032-2-272; Indonesia) were purchased from Yaksudang Pharmaceutical Co. (Seoul, Korea). The plants were certified by Donghun Lee and voucher specimens (No. 2009150006) were entrusted to the College of Korean Medicine at Gachon University. The dried fruit was extracted using a reflux apparatus with 30% EtOH at 85°C for 3 h. The extract was concentrated, filtered under decreased pressure, and lyophilized to yield a powder (extraction yield: 8.26%) (Lee et al., 2021; Jo et al., 2023a).

### 2.3 High performance liquid chromatographic analysis of PLE

For the component analysis of PLE, high performance liquid chromatography (HPLC) was performed using a 1,100 series HPLC system (Agilent, United States), and the analysis conditions are shown in Table 1. PLE (10 mg) was diluted with methanol (1 mL) and sonicated for 10 min. Samples were filtered using a 0.45-μm syringe filter (Waters Corp., United States) (Lee et al., 2020; Jo et al., 2023a).

**TABLE 1:** Analytical conditions of PLE.

	Condition 1	Condition 2
Column	Luna C18 column (250 mm × 4.6 mm, 5 μm; Phenomenex, United States)	Triart C18 column (150 mm × 4.6 mm id, 5 μm) (YMC-PACK ^®^, Japan)
Mobil phase	MeOH:Water (0.1% acetic acid) at 70:30%	Acetonitrile:Water (50:50%)
Flow rate	1.0 mL/min	1.0 mL/min
Injection volume	10 µL	10 µL
Detection wavelength	338 nm	325 nm
Temperature	30°C	30°C

### 2.4 Cell culture

Mouse leukemia cells derived from RAW264.7 macrophages were purchased from the American Type Culture Collection (ATCC, TIB-71 ™). Cells were incubated at 37°C and 5% CO_2_ in complete media (DMEM+10% FBS+100 U/mL penicillin–streptomycin; Gibco™ Inc., United States) (Guo et al., 2019; Lee et al., 2020).

### 2.5 NO production and cell toxicity analysis

RAW264.7 cells were seeded and grown at 37°C and 5% CO_2_ for 24 h. RAW264.7 cells were cultured in 1 μg/mL dexamethasone (Sigma, United States), 10–300 μg/mL PLE and 500 ng/mL lipopolysaccharide (LPS) for 24 h. Then, cell supernatants were mixed with Griess reagent (1:1 ratio), and the NO concentration was measured at 540 nm (Lee et al., 2019; Jo et al., 2020). Cell viability was analyzed using Ez-Cytox reagent (DoGenBio, Korea) according to the manufacturer’s protocols (Yu et al., 2020; Jo et al., 2023a). This experiment was repeated triplicated.

### 2.6 Animals

Male Sprague–Dawley (SD) rats and ICR mice were provided by DBL Co., Ltd. (DBL, Korea). The rats and mice were housed in open-top cages (W260 × D420 × H180 mm, W200 × D260 × H130 mm, Jeung Do Bio&Plant Co. Ltd., Korea) with SAFE^®^ 40 bedding (SAFE Inc., France). For at least 7 days prior to the experiment, animals were acclimatized to regular laboratory settings (55% ± 10% humidity, 22°C ± 2°C, and a 12-h light–dark cycle). The animals were allowed free access to food and water. All the procedures were approved by the Gachon University Center of Animal Care and Use (GIACUC-R2020028).

### 2.7 Acetic acid (AA)-induced writhing test

ICR mice (35 ± 5 g) were randomly separated into five groups (n = 8 per group): control, ibuprofen, PLE 200 and 600 mg/kg. Mice were injected intraperitoneally on the right side of the abdominal midline with 10 mL/kg of 0.7% AA in 0.9% saline using a 23-gauge needle and a 1 mL syringe (Korea Vaccine Co., Ltd, Korea). The number of writhes was measured for 10 min, starting from when the AA solution was administered. Writhing is defined as abdominal muscle contraction with elongation of the body and rear limbs. Thirty minutes before AA injection, the mice were administered 200 mg/kg ibuprofen (Sigma, United States) and 200 or 600 mg/kg PLE. The analgesic response was characterized by a significant reduction in writhing in the experimental group compared to that of the control group (Yin et al., 2016; Lee et al., 2020). The experimental data was cross-checked by four people. Mice were sacrificed using CO_2_ after the counting period for the writhing was completed.

### 2.8 OA induction by monosodium iodoacetate (MIA) injection

SD Rats (190–210 g) were randomly divided into five groups (n = 9 per group, NT; n = 3) as follows: non-treated (NT), MIA, indomethacin, and PLE 100 and 300 mg/kg groups. Animals were anesthetized using isoflurane with N_2_O and O_2_ (7:3), and were injected with 50 µL sterile 0.9% saline with 40 mg/mL of MIA (cat no. I2512-25, Sigma, United States) into the right knee joint, except for the rats in the NT group (Yin et al., 2016; Jo et al., 2023a).

### 2.9 Diet

Experimental rats were treated as follows: NT and MIA rats were fed a basic diet (AIN-93G; Saeronbio., Inc, Korea), indomethacin-treated rats were fed an AIN-93G diet including 0.003% indomethacin (final dose: 3 mg/kg; Sigma, United States), and both PLE groups were fed an AIN-93G diet including 0.11% and 0.33% of PLE (final dose: 100 and 300 mg/kg). After the induction of OA using MIA, a diet of 10 g food per 100 g body weight was provided daily for 24 days.

### 2.10 Weight-bearing measurement of hind leg

An Incapacitance Meter Tester 600 series 8 (IITC Life Science Inc., United States) was used to monitor weight-bearing from OA-induction until 24 days after MIA injection. The strength recorded for each limb was averaged over 10 s (Sahin et al., 2023). The experimental data was cross-checked by two experimenters. The following equation was used to determine the percentage of weight distributed in the right rear limb on the treated side:

$$\text{weight bearing ratio}\left(\%\right)=\left(\text{weight on right hind limb}/\text{weight on left and right hind limb}\right)\times 100$$

### 2.11 Cartilage degradation evaluation

After the mice were sacrificed, the right knees were disarticulated and imaged for macroscopic scoring. The erosion of the articular cartilage was graded according to the macroscopic scoring system. Macroscopic scores are shown in Supplementary Table S4 (Tian et al., 2021).

### 2.12 Serum analysis of the OA-induced model

Blood was drawn from the abdominal vein of rats 24 days after OA induction and allowed to clot for 30 min. After 10 min of centrifugation at 4,000 rpm, the serum was divided and kept at −70°C. To measure cytokine in the serum, a multiplex assay for IL-6 and TNF-α was performed using a Premixed-MultiAnalyte Kit (R&D Systems, United States). All multiplex assays were performed according to the manufacturer’s instructions (Jo et al., 2020; Kimmerling et al., 2020). This experiment was repeated triplicated.

### 2.13 Quantitative real-time polymerase chain reaction (qRT-PCR) analysis

Total RNA was extracted from OA-induced cartilage tissues (articular cartilage and meniscus) and LPS-stimulated RAW264.7 cells using the AccuPrep^®^ universal RNA Extraction Kit (Bioneer corp., Korea) and then reverse-transcribed into cDNA using the CycleScript™ RT Pre&Master Mix (Bioneer, Korea), following the manufacturer’s protocol. mRNA expression was quantified using 2X-GreenStar qPCR MasterMix (Bioneer, Korea) (Lu et al., 2018; Lee et al., 2020). This experiment was repeated triplicated. The primer sequences are listed in Supplementary Tables S1, S2.

### 2.14 Protein expression analysis

Western blotting (WB) was performed to examine the protein expression levels of F2R, F3, IL-17A, MMP-1, MMP-2, MMP-9, PTGS2, and GAPDH. Total protein was extracted from OA-induced cartilage using RIPA buffer (CST Inc., United States) and a cOmplete Protease Inhibitor Cocktail (Sigma, United States) in a homogenizer (Nissei Corp., Japan). Protein samples (10 μg) were placed onto Mini-PROTEAN TGX Precast Gel (BioRad Laboratories, Inc., United States), and the extracted proteins were transferred onto PVDF membranes for 1 h using the Mini Trans-Blot Cell (BioRad, United States) at 100 V. To inhibit non-specific antibody binding, membranes were washed using EveryBlot blocking buffer (BioRad, United States) for 15 min at RT. The primary antibodies (F2R, F3, IL-17A, MMP-1, MMP-2, MMP-9, PTGS2, and GAPDH) were applied to react for 24 h at 4°C. CST Inc., BOSTER Inc., Proteintech Inc., and Abcam Inc. supplied the antibodies (cat No. A03352-1, M00342, ab214588, 10371-2-AP, M00286-3, M00139, ab179800 and 2118). The membrane was probed with a secondary antibody for 1 h at RT before reacting with Clarity Western ECL Substrate (Bio-Rad) solution. WB was performed using an Azure 280 (Azure Biosystems, United States). This experiment was repeated triplicated.

### 2.15 Statistics

GraphPad Prism^®^ 5.0 (GraphPad Software, San Diego, United States) was used for statistical analysis, including 1-way ANOVA with Dunnett’s *post hoc* test. 2-way ANOVA with Tukey’s multiple comparisons test was used to compared with doses and the treatment group at the different times. Significance level was set at *p* < 0.05, and measurements data was as mean ± standard error of the mean.

## 3 Results

### 3.1 Network analysis prediction of PL against OA

#### 3.1.1 Screening of target genes related to the active compounds of PL and target genes of OA

**FIGURE 1:**
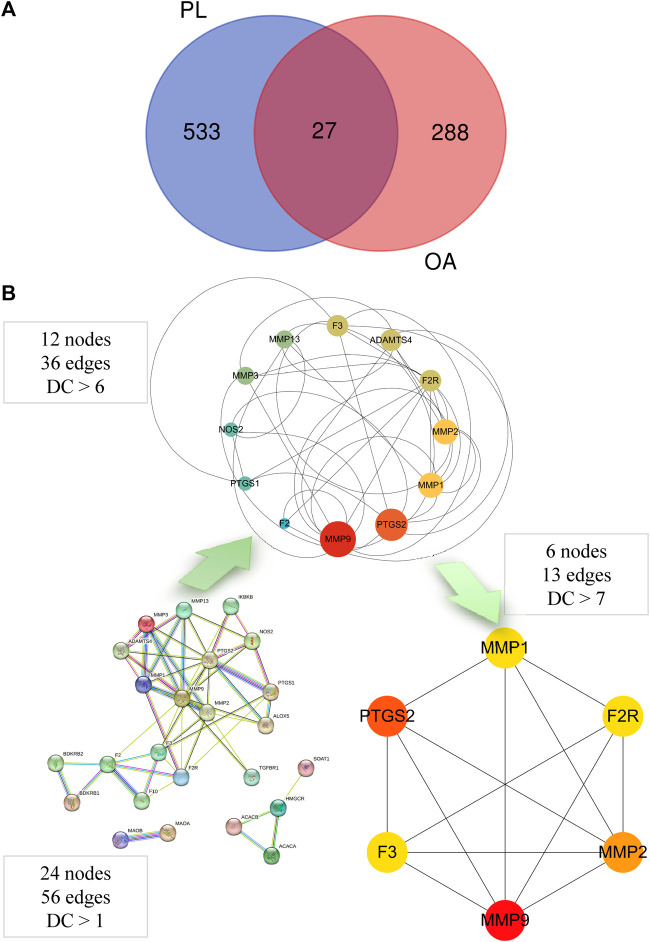
**(A)** Venn diagram of potential targets for PL in the treatment of OA. PL: *Piper longum* L. [Piperaceae]; OA: osteoarthritis. **(B)** PPI network construction sequence of PL-OA genes by degree centrality. GO and KEGG analysis.

After collecting the components of PL from five databases and screening them according to the criteria of OB ≥ 30% and DL ≥ 0.18, 28 potential active compounds were identified (Table 2). The Swiss TargetPrediction database was used to search for targets of each compound, and a total of 533 targets were obtained, excluding duplicates (Supplementary Table S3). A total of 288 OA targets with a relevance score ≥10 were obtained from the Genecard database. Based on the Venn diagram of targets in PL and OA, 27 overlapping gene targets were considered potential targets for PL against OA (Figure 1A).

**TABLE 2:** Potential active compounds of *Piper longum* L. [Piperaceae].

Pubchem ID	Compound name	Structure	OB (%)	DL
72,307	sesamin	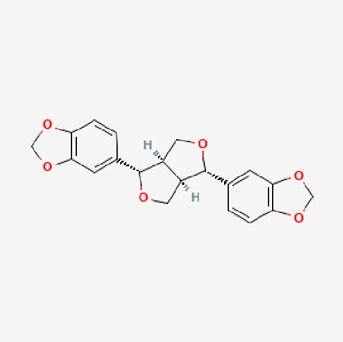	56.55	0.83
101,689	Pisatin	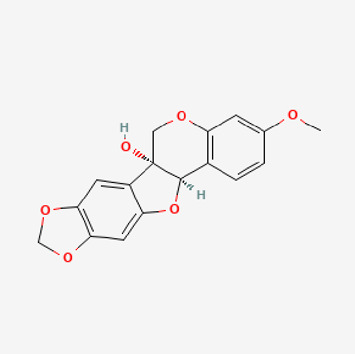	88.05	0.64
124,416	Trijuganone B	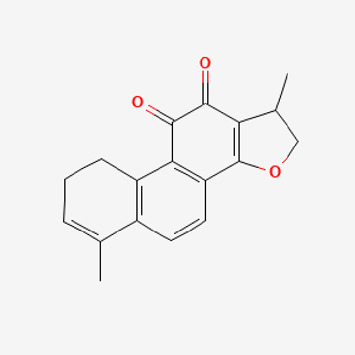	38.75	0.36
267,400	Laurotetanine	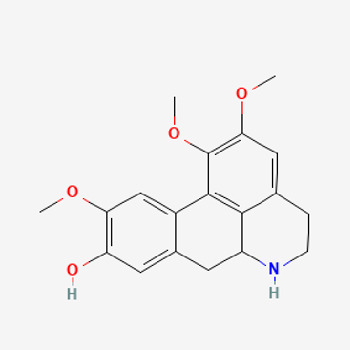	55.41	0.51
441,737	Hypaconitine	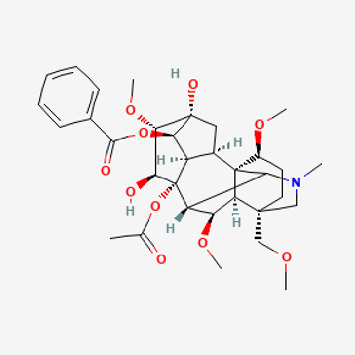	31.39	0.26
532,276	N-(2,5-dimethoxyphenyl)-4-methoxybenzamide	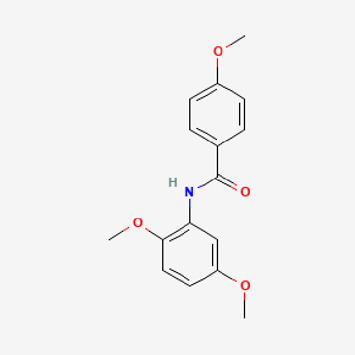	60.7	0.18
637,858	Piperlongumine	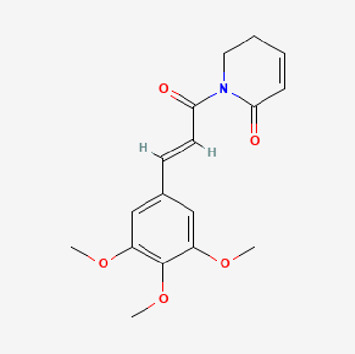	96.65	0.24
638,024	piperine	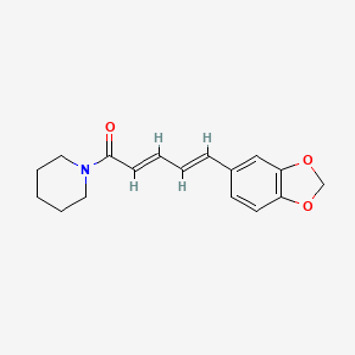	42.52	0.23
643,764	cis-Piplartine	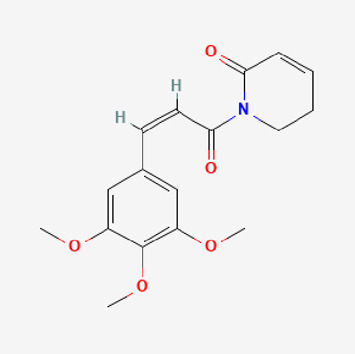	96.65	0.24
5,315,472	Bisdemethoxycurcumin	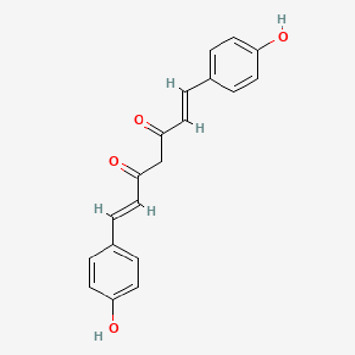	77.38	0.26
5,320,621	Piperlonguminine	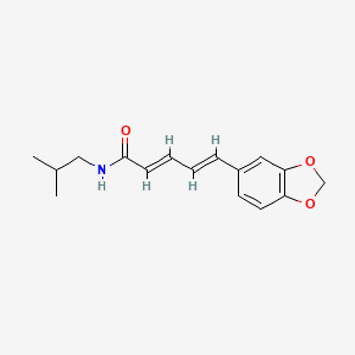	30.71	0.18
5,372,162	Pipercide	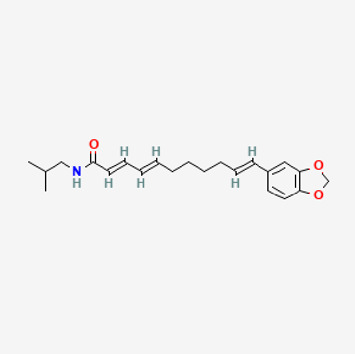	42.72	0.43
6,439,947	Dehydropipernonaline	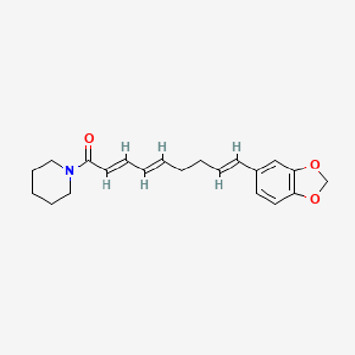	47.73	0.41
6,441,067	N-Isobutyl-2,4-icosadienamide	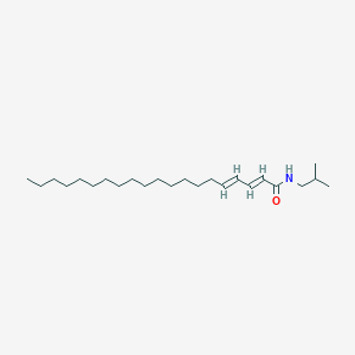	38.86	0.32
6,442,405	Guineensine	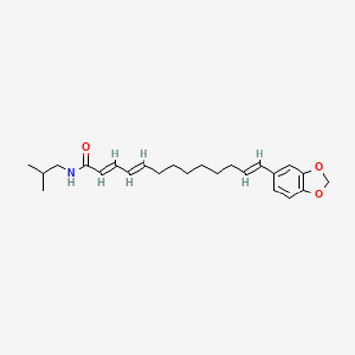	42.64	0.53
6,453,083	(E,E,E)-11-(1,3-Benzodioxol-5-yl)-N-(2-methylpropyl)-2,4,10-undecatrienenamide	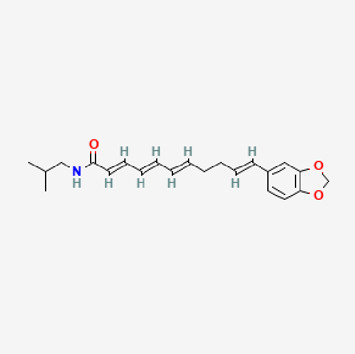	42.72	0.43
7,299,790	(−)-Epieudesmin	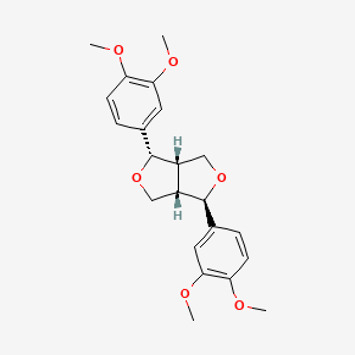	52.35	0.62
9,974,595	pipernonaline	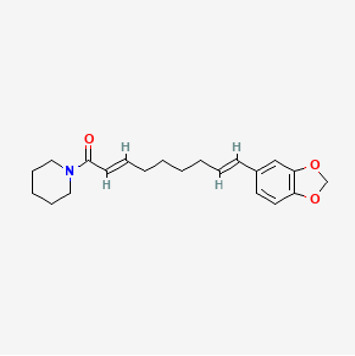	51.32	0.41
11,012,859	Retrofractamide A	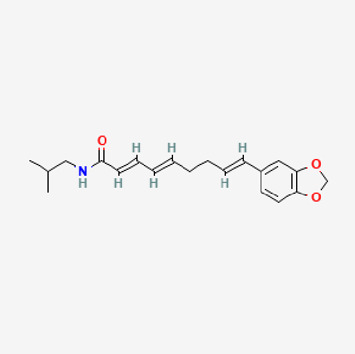	65.9	0.33
11,870,467	ZINC03982454	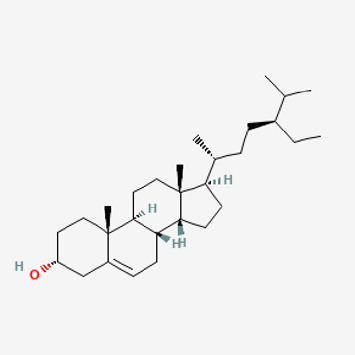	36.91	0.76
44,453,654	Piperundecalidine	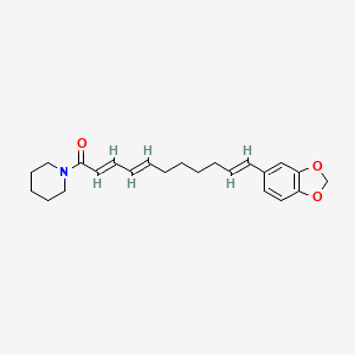	29.96	0.52
78,358,503	3-Deoxyaconitine	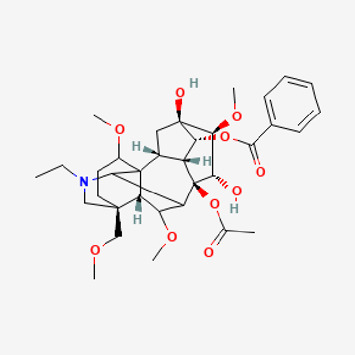	30.96	0.24
90,472,536	(2E,4E)-5-(1,3-benzodioxol-5-yl)-N-[(E)-10-methylundec-5-enyl]penta-2,4-dienamide	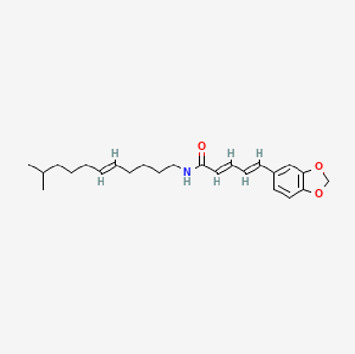	44	0.51
71,448,929	Ignavine	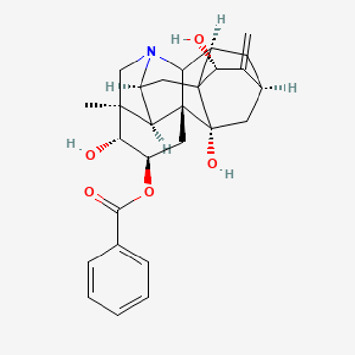	84.08	0.25
131,750,975	(E,E,E)-Sylvatine	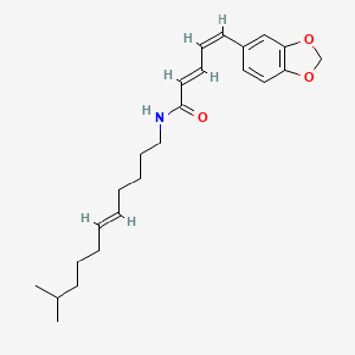	44	0.51
131,752,411	(2E,8E)-Piperamide-C9:2	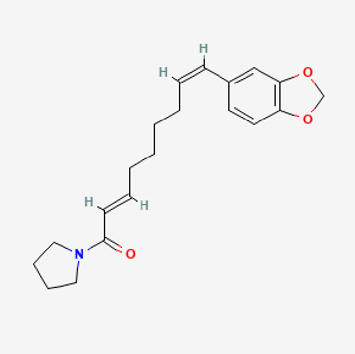	49.43	0.36
134,884,778	5-[(E)-undec-1-enyl]-1,3-benzodioxole	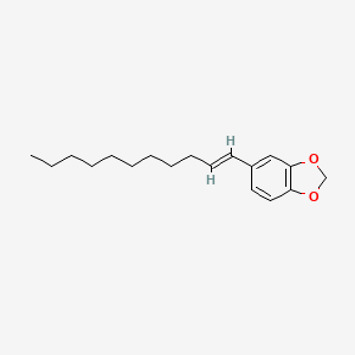	47.97	0.18
155,167,294	(2E,4E,8E)-N-(2-methylpropyl)icosa-2,4,8-trienamide	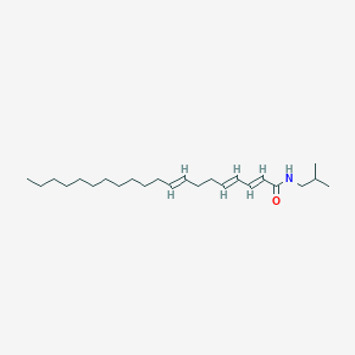	44.48	0.32

OB, oral bioavailability; DL, drug likness.

#### 3.1.2 Protein–protein interaction (PPI) network construction

After importing the identified common targets into the STRING 11.5 platform, a PPI network model (minimum required interaction score:0.4) was generated by restricting the organism to *H. sapiens*. Three genes (*MTAP*, *PDK1*, and *SLC22A12*) that did not interact with the other targets were excluded from the PPI network. We identified 56 nodes with 56 edges and an average node degree of 4.67. Cytoscape software was used to visualize and analyze the network by calculating centrality metrics. A higher centrality value indicates a more important role in the network. Finally, six key gene targets that satisfied a high degree centrality of >7.0, corresponding to the top 25% centrality were identified: *F2R*, *F3*, *MMP1*, *MMP2*, *MMP9*, and *PTGS2* (Figure 1B).

#### 3.1.3 Construction of the D-C-T-P-D network

**FIGURE 2:**
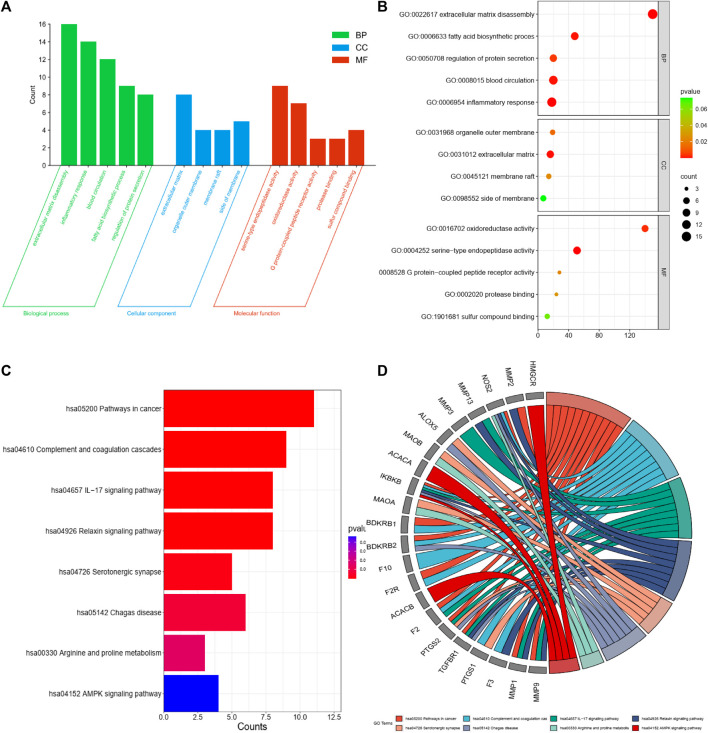
**(A)** Top 5 gene ontology (GO) enrichment terms for biological process, cellular components, and molecular functions. **(B)** Bubble plot of GO enrichment results. **(C)** Horizontal bar plot of Kyoto Encyclopedia of genes and genomes (KEGG) pathway enrichment analysis illustrating eight enriched pathways. **(D)** GO chord diagram of KEGG pathway analysis.

**FIGURE 3:**
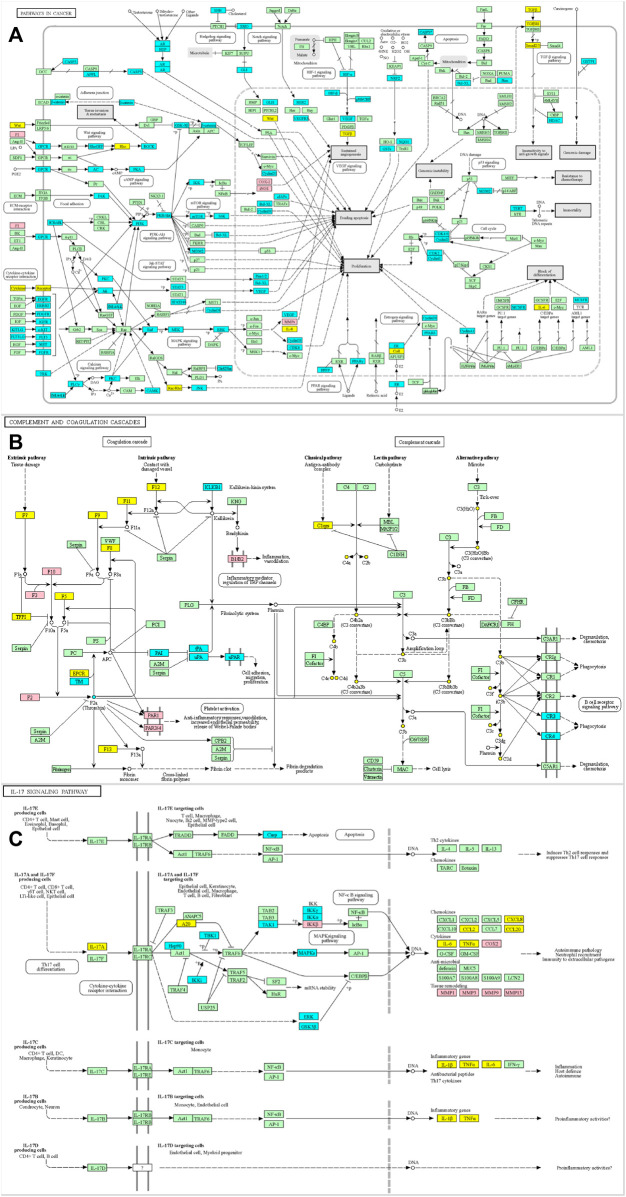
**(A)** Pathways related to cancer were colored using the KEGG mapper. **(B)** The complement and coagulation cascade were colored using the KEGG mapper. **(C)** The IL-17 signaling pathway was colored using the KEGG mapper. In all figures, pink represents PL compounds that alleviate OA, cyan colors represent targets of PLs not involved in OA treatment, and yellow colors represent other OA-related targets in each pathway.

Functional GO analysis was used to comprehensively identify the pharmacodynamic properties of the potential key compounds in PL. This analysis was performed by importing common targets into the Metascape platform. The identified BPs included extracellular matrix degradation, inflammatory response, blood circulation, fatty acid biosynthetic processes, and regulation of protein secretion (Figures 2A, B). A total of 25 items were identified for MF, including serine-type endopeptide activity, oxidoreductase activity, G protein-coupled peptide receptor activity, protease binding, and sulfur compound binding (Figures 2A, B). Fifteen items were identified as CCs, including the extracellular matrix, organelle outer membrane, membrane raft, and side of the membrane (Figures 2A, B). The KEGG pathway enrichment analysis revealed that PL was mainly involved in 37 pathways (Figures 2C, D). The main pharmacological mechanisms of PL in OA have been found to be related to pathways in cancer, complement and coagulation cascades, and the IL-17 signaling pathway. The three major pathway targets were colored using a KEGG mapper to further illustrate the mechanism of action (Figures 3A–C).

#### 3.1.4 Construction of the D-C-T-P-D network

**FIGURE 4:**
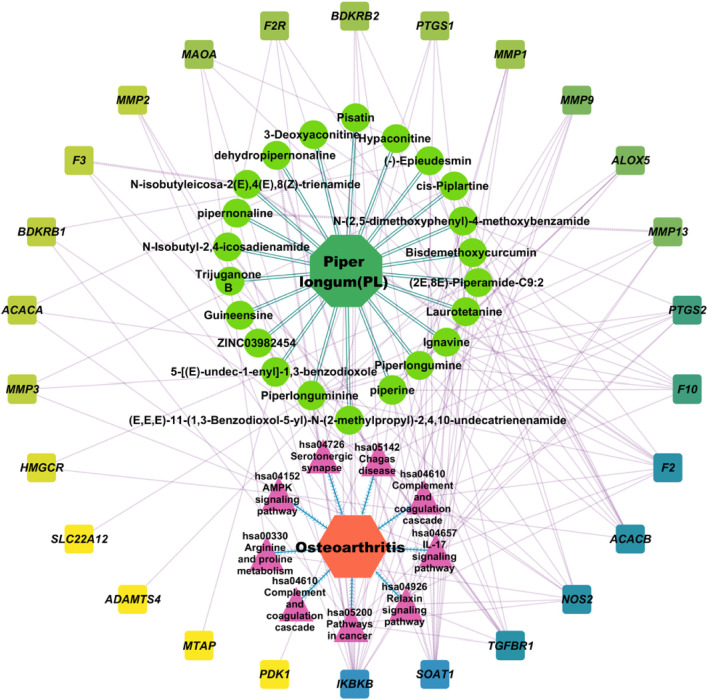
The therapeutic mechanisms of PL in OA are represented by the drug-target-compound-pathway-disease network. Drugs are represented by green octagons, key compounds by lime green circles, targets by squares that range in color from blue to yellow depending on degree centrality, top eight pathways of action by pink triangles, and OA by orange hexagons.

A D-C-T-P-D network summarizing the therapeutic mechanism of PL against OA was constructed using Cytoscape (Figure 4). The network consisted of 59 nodes and 162 edges, indicating that, even as a single herb, PL acts in a multi-compound and multi-target manner against OA. The centrality of the active compounds was evaluated using network analysis. The four compounds with the highest degree of centrality in the network were piperlonguminine (11), piperlongumine (8), piperine (8), and ignavine (8), all of which were considered core compounds involved in the action of PL against OA.

#### 3.1.5 Molecular docking

**FIGURE 5:**
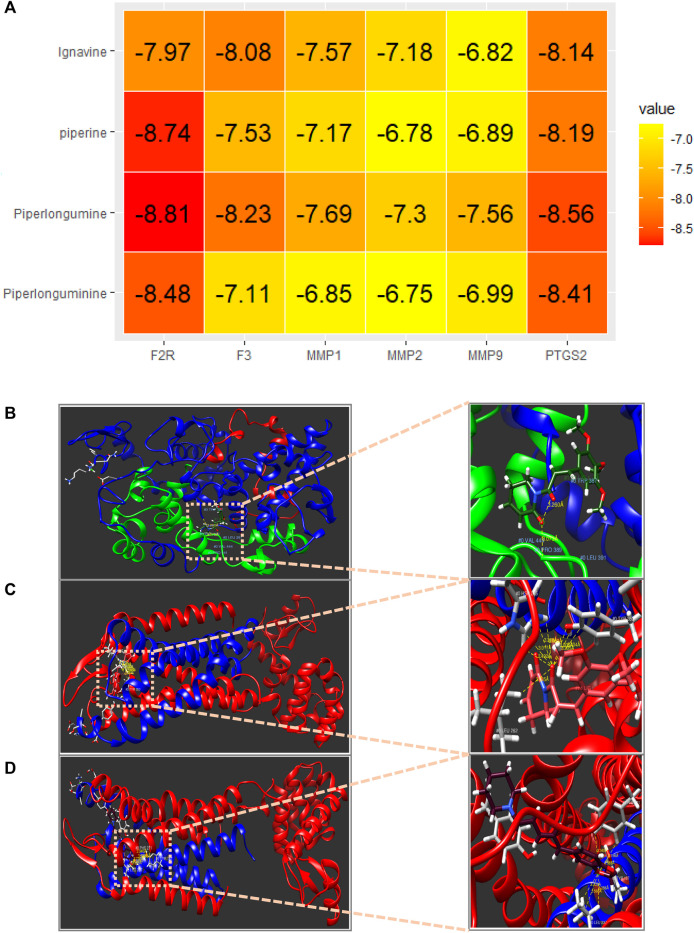
**(A)** The binding energy between the six most important targets and the core compounds was calculated via molecular docking models. **(B)** Piperlongumine and PTGS2, binding energy = −8.56 kcal/mol. **(C)** Piperlongumine and F2R, binding energy = −8.81 kcal/mol. **(D)** Piperine and F2R, binding energy = −8.74 kcal/mol.

Finally, a molecular docking evaluation was performed to predict the binding potential between the four core compounds (piperlonguminine, piperlongumine, piperine, and ignavine) and six important targets (F2R, F3, MMP1, MMP2, MMP9, and PTGS2) selected by the PPI network analysis using the Swiss Dock website (Figure 5A). According to previous studies, a binding affinity of −4.25 kcal/mol means that the two molecules bind with average performance, −5.0 kcal/mol means good binding, and −7.0 kcal/mol means strong binding energy (Saikia and Bordoloi, 2019). All docking results were close to or higher than −7.0 kcal/mol, and piperlongumine showed the strongest binding energy (−8.81 kcal/mol) for F2R. Piperine exhibited strong binding energy values of −8.74 kcal/mol and −8.19 kcal/mol for F2R and PTGS2, respectively. Piperlongumine also showed a strong binding energy (−8.48 kcal/mol) for F2R, and binding energies higher than −7.0 kcal/mol for all other targets. In addition, piperlonguminine, piperine, and ignavine exhibited good overall binding energies. Figures 5B–D show representative 3D binding conformations corresponding to < −8.5 kcal/mol.

### 3.2 Network analysis prediction of PL against OA HPLC analysis

**FIGURE 6:**
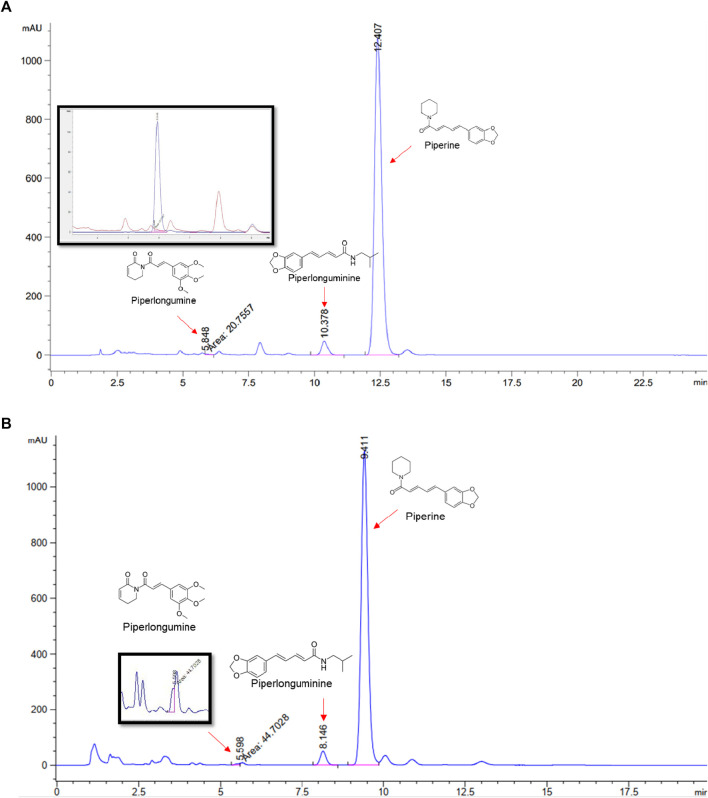
High-performance liquid chromatography (HPLC) chromatogram of *Piper longum* extract (PLE) at 338 nm. *X*-axis is retention time; *Y*-axis is absorbance unit. **(A)** Piperlongumine, piperlonguminine, and piperine retention times = 6.347 min, 10.315 min, and 12.332 min, respectively. A Luna C18 column (250 mm × 4.6 mm, 5 μm; Phenomenex, United States) was used for chromatic separation at 30°C. **(B)** Wavelength was 232 nm: Piperlongumine, Piperlonguminine, and Piperine Retention time = 5.598 min, 8.146 min, and 9.411 min, respectively. A Triart C18 column (150 mm × 4.6 mm id, 5 μm) (YMC-PACK^®^, Japan) is used for chromatic separation at 30°C.Effects of PLE on cell viability in stimulated RAW264.7 cells stimulated by LPS.

Piperlongumine, piperlonguminine, and piperine were identified in PLE by HPLC-UV. Fist, we measured the conditions; 338 nm, A Luna C18 column (250 mm × 4.6 mm, 5 μm; Phenomenex, United States). The contents of piperlongumine, piperlonguminine, and piperine in PLE were 0.92 mg/g, 3.88 mg/g, and 89.34 mg/g, respectively. The retention times of piperlongumine, piperlonguminine, and piperine were 6.347 min, 10.315 min, and 12.332 min, respectively (Figure 6A). Next, we analyzed another condition; 232 nm, A Triart C18 column (150 mm × 4.6 mm id, 5 μm) (YMC-PACK^®^, Japan) (Figure 6B). The contents of piperlongumine, piperlonguminine, and piperine in PLE were 0.21 mg/g, 2.52 mg/g, and 34.78 mg/g, respectively. The retention times of piperlongumine, piperlonguminine, and piperine were 5.598 min, 8.146 min, and 9.411 min. The HPLC chromatogram of the analysis and the chemical structure of the component compounds are shown in Figure 1.

**FIGURE 7:**
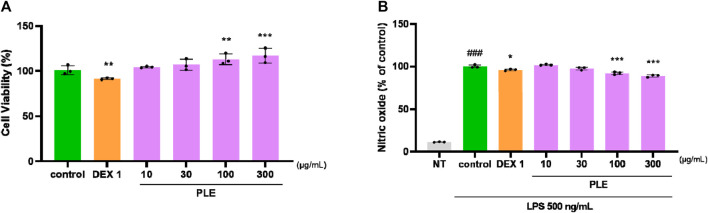
Effects of PLE on **(A)** cell viability and **(B)** NO production in stimulated RAW264.7 cells by LPS. Cell were included with PLE (10–300 μg/mL) and LPS (500 ng/mL) for 24 h ###*p* < 0.001 vs. NT, *p < 0.05 vs. control, **p < 0.01 vs. control, ***p < 0.005 vs. control. NT: non-treated, DEX 1: dexamethasone 1 μg/mL, LPS: lipopolysaccharide.

PLE demonstrated anti-inflammatory effects in LPS-stimulated RAW264.7 cells by reducing nitric oxide (NO). PLE did not exhibit cytotoxicity in RAW264.7 cells up to a concentration of 300 μg/mL (Figure 7A). PLE downregulated LPS-induced NO production in a dose-dependent manner (Figure 7B).

### 3.3 Effects of PLE on inflammatory responses in LPS-stimulated RAW264.7 cells

**FIGURE 8:**
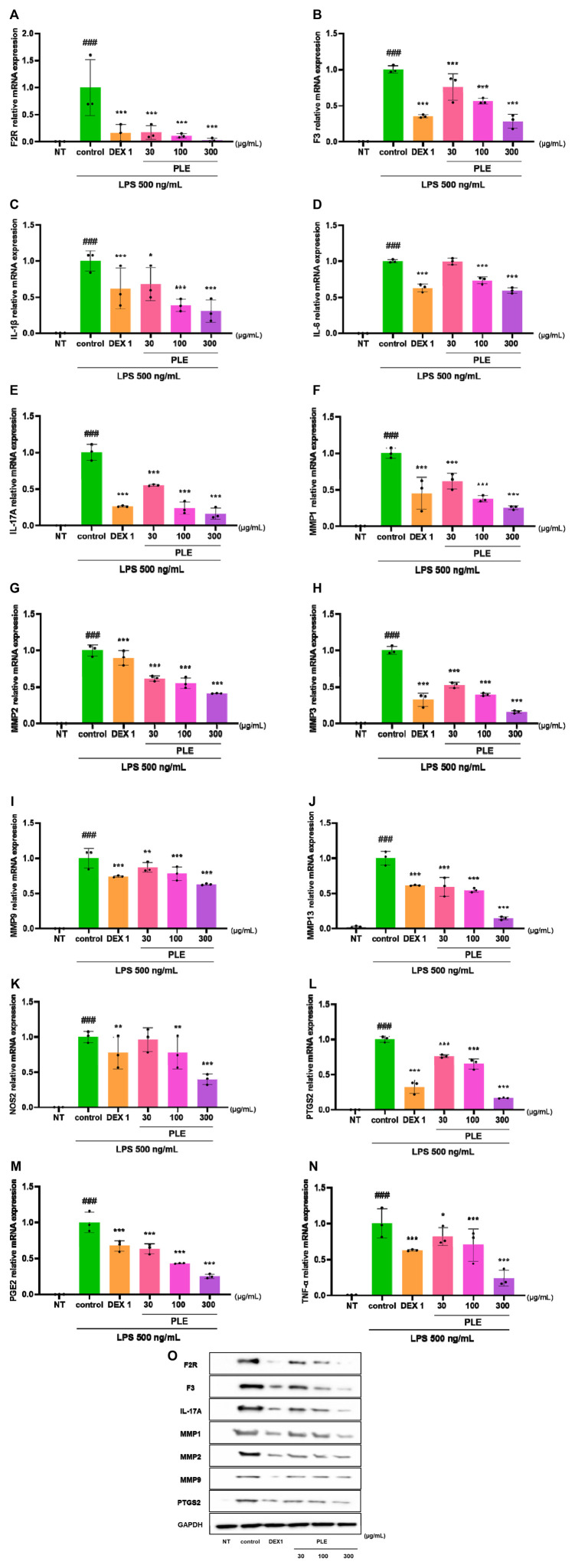
Effects of PLE on **(A–N)** qRT-PCR analysis **(O)** Western blotting in LPS-stimulated RAW264.7 cells. Cells were treated with PLE (30, 100, and 300 μg/mL) and LPS (500 ng/mL) for 24 h ###*p* < 0.001 vs. NT, *p < 0.05 vs. control, **p < 0.01 vs. control, ***p < 0.005 vs. control. NT: non-treated, DEX 1: dexamethasone 1 μg/mL.

Anti-inflammatory effects of PLE on LPS-stimulated RAW264.7 cells were evaluated through qRT-PCR and WB. As shown in Figures 8A–N; Supplementary Table S11, PLE and indomethacin decreased the mRNA expression levels of F2R, F3, IL-1β, IL-6, IL-17A, MMP-1, MMP-2, MMP-3, MMP-9, MMP-13, NOS2, PTGS2, PGE2, and TNF- α. PLE (300 μg/mL) showed similar effects to those of dexamethasone, and the anti-inflammatory effect of PLE was dose-dependent. WB was performed to evaluate the anti-inflammatory effects of PLE on LPS-activated RAW264.7 cells. PLE treatment suppressed the protein expression levels of F2R, F3, IL-17A, MMP-1, MMP-2, MMP-9, and PTGS2, as well as pro-inflammatory cytokines and mediators in LPS-stimulated RAW264 cells. As shown in the WB image, the expression of F2R, F3, IL-17A, MMP-1, MMP-2, MMP-9, and PTGS2 was decreased by PLE in a dose-dependent manner. Remarkably, 300 μg/mL PLE showed stronger anti-inflammatory effects than those of the positive control (Figure 8O).

### 3.4 Effect of PLE on AA-induced writhing

**FIGURE 9:**
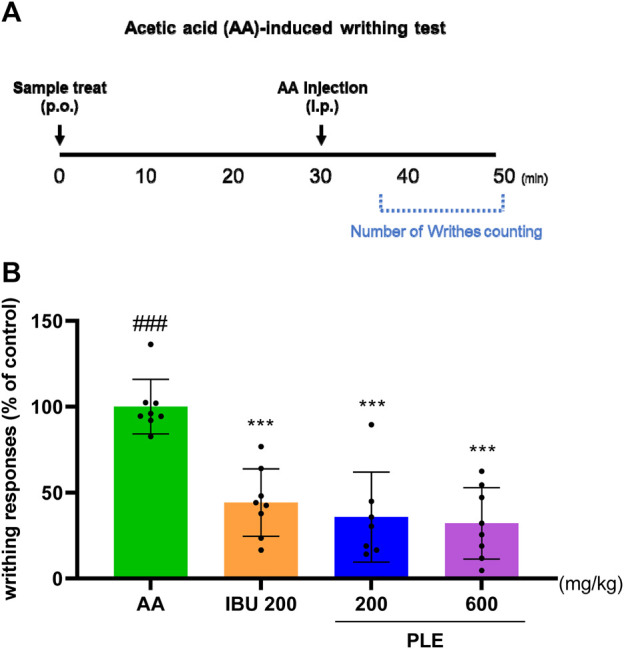
Analgesic effect of PLE in on nociceptive responses in the acetic acid (AA)-induced-writhing test **(A)** Timeline for the AA-induced writhing test. The writhing number of AA-induced ICR mice. **(B)**After 30 min of oral treatment, mice were intraperitoneally injected with 0.7% AA before counting for 10 min (eight mice per group); ###*p* < 0.001 vs. IBU 200, **p < 0.05 vs. AA, ***p < 0.001 vs. AA. IBU: ibuprofen 200 mg/kg.

The writhing test in AA-induced mice was used to investigate the analgesic effects of PLE. The analgesic effect of PLE was observed in AA-induced mice via writhing responses. For the 10-min counting period, the average writhing number in the AA group was 100. When compared to the control, the PLE treatment reduced the amount of writhing. The average writhing number of the mice fed 600 PLE was 32.14, which was lower than that of the positive AA (44.17). This result demonstrated the analgesic effects of PLE (Figure 9; Supplementary Table S7).

### 3.5 Effects of PLE on the weight-bearing index (WBI) of OA-induced rats

**FIGURE 10:**
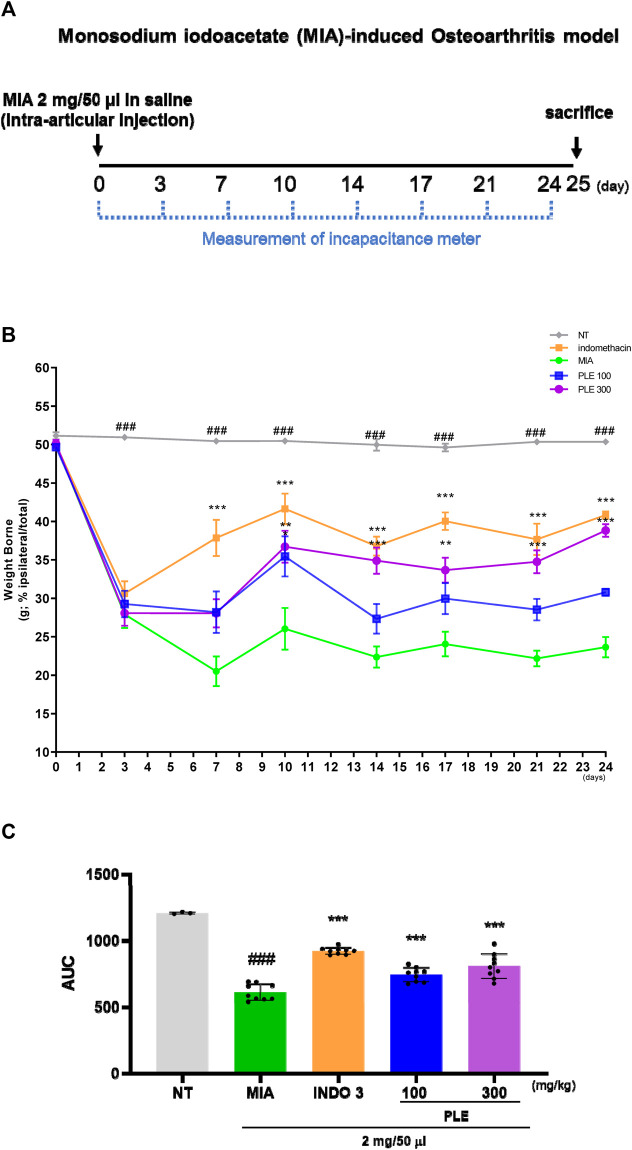
The effects of PLE on weight-bearing index (WBI) of hind legs in monosodium-iodoacetate (MIA) induced rats. **(A)** Timeline for the MIA-induced OA model. **(B)** WBI of MIA rats at 0–24 days treated with 100 and 300 mg/kg PLE or 3 mg/kg indomethacin and **(C)** incapacitance meter analysis area under the curve (AUC). Tukey’s multiple comparison test after 2-way ANOVA: ###*p* < 0.001 vs. NT, *p < 0.05 vs. MIA, **p < 0.01 vs. MIA, ***p < 0.001 vs. MIA. INDO 3: indomethacin 3 mg/kg.

In OA-induced rats, WBI of the hind legs is often measured to assess the analgesic effects of natural compounds on OA. WBI between the left and right legs was recorded for 24 days after OA induction using MIA. As shown in Figure 10A, WBI in the MIA rats were significantly reduced on day 7 and remained lower afterwards, compared with that of the NT rats. Notably, WBI was significantly improved in PLE-treated rats. Particularly, 300 mg/kg PLE-treated rats recovered to a level comparable to that of the group treated with 3 mg/kg indomethacin (Figures 10B, C; Supplementary Table S5).

### 3.6 Effects of PLE on knee joint erosion in OA-induced rats

**FIGURE 11:**
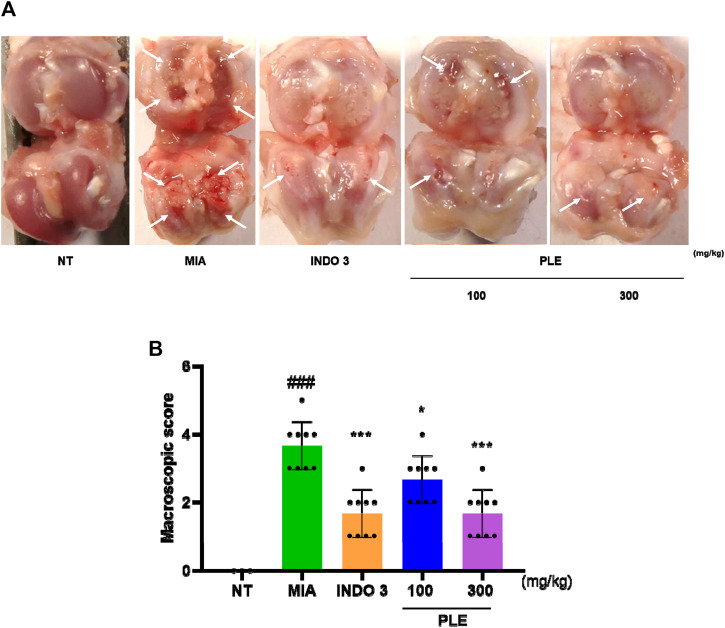
Representative images of articular cartilage in OA-induced rats treated with MIA. **(A)** representative picture showed cartilage degradation. **(B)** Macroscopic score. MIA rats were treated with 3 mg/kg indomethacin and 100 or 300 mg/kg PLE. NT: non-treated, INDO 3: indomethacin 3 mg/kg.

Representative images of the knee joints of each experimental group indicated that PLE prevented cartilage degradation induced by MIA injection. In contrast to the cartilage of the MIA rats, which was less smooth and more damaged in some places, the knee joints of the NT rats were glossy and smooth. Rats treated with PLE and indomethacin showed significant recovery from cartilage damage caused by MIA. The recovery of cartilage erosion in PLE-treated rats was comparable to that in indomethacin-treated rats (Figure 11A). Characteristics of OA, such as cartilage erosion on the side of the femoral condyles, were found in the MIA group. The cartilage injury worsened over time. However, based on gross appearance, the PLE group showed less bone degradation and cartilage erosion than the MIA group during the same time period. When compared to the MIA group, the macroscopic score of the PLE groups was lowered by 55.17% (Figure 11B; Supplementary Table S6).

### 3.7 Effects on inflammatory cytokines in the serum of OA inducted rat model

**FIGURE 12:**
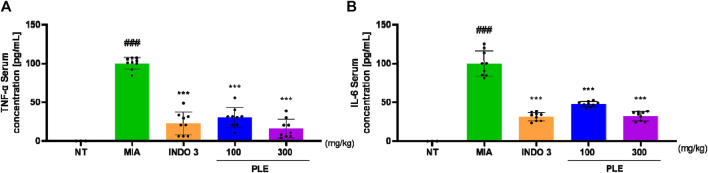
Inflammatory cytokine levels in the sera of OA-induced rats. Rats were treated with 100 and 300 mg/kg of PLE for 24 d ###*p* < 0.001 vs. NT, **p < 0.05 vs. MIA, ***p < 0.001 vs. MIA. NT: non-treated, INDO 3: indomethacin 3 mg/kg.

After isolating blood sera from each experimental group, the levels of TNF-α and IL-6 were assessed. Serum concentrations of TNF-α and IL-6 were significantly lower in PLE-treated rats than in MIA rats, in a dose-dependent manner. PLE-treated rats (300 mg/kg body weight) showed reduced cytokine levels, consistent with those of the positive control (Figure 12; Supplementary Table S9).

### 3.8 Effects of PLE on cytokine responses in the knee cartilage tissue of OA-induced rats

**FIGURE 13:**
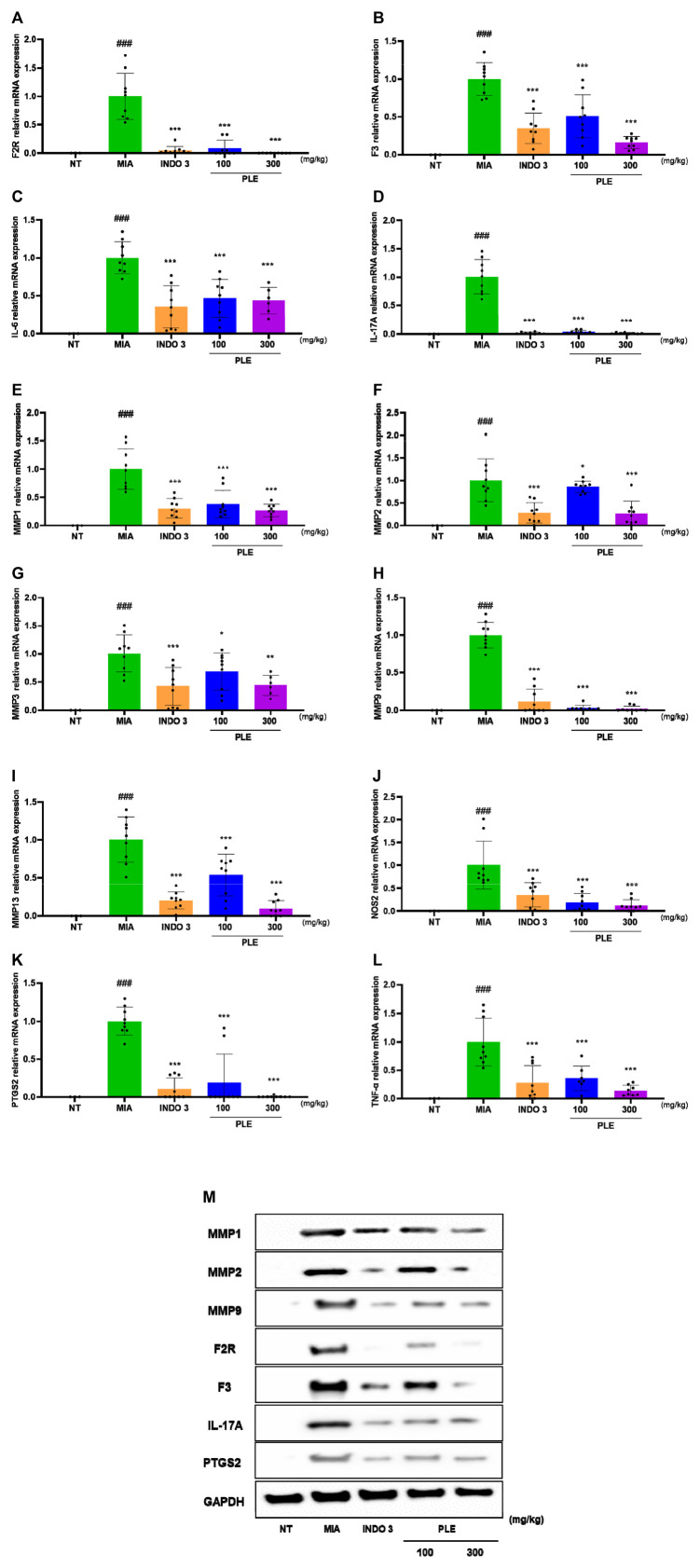
Cytokine levels in the knee cartilage tissue of 100 and 300 mg/kg PLE-treated rats. **(A–L)** mRNA expression of F2R, F3, IL-1β, IL-6, IL-17A, MMP-1, MMP-2, MMP-3, MMP-9, MMP-13, NOS2, PTGS2, PGE2, and TNF-α determined by qRT-PCR. **(M)** WB analysis of the protein expression of F2R, F3, IL-17A, MMP-1, MMP-2, MMP-9, and PTGS2. ###*p* < 0.001 vs. NT, **p < 0.05 vs. MIA, ***p < 0.001 vs. MIA. NT: non-treated, INDO 3: indomethacin 3 mg/kg.

The analysis of F2R, F3, IL-1β, IL-6, IL-17A, MMP-1, MMP-2, MMP-3, MMP-9, MMP-13, NOS2, PTGS2, PGE2, and TNF-β mRNA levels (Figures 13A–L) in OA-induced rats determined that PLE-treated rats had significantly reduced knee cartilage tissue compared with that of the MIA rats. The anti-inflammatory effects of PLE were dose-dependently comparable to those of dexamethasone at 300 mg/kg. WB analysis indicated that PLE inhibited F2R, F3, IL-17A, MMP-1, MMP-2, MMP-9, and PTGS2 in MIA rats (Figure 13M; Supplementary Table S10).

## 4 Discussion

In this study, we used a network analysis approach to predict the major compounds and target genes and pathways of PL that are expected to play a key role in attenuating OA symptoms and progression. We then investigated these predictions by designing *in vivo* and *in vitro* experiments that mimicked the pathophysiology of OA. From a network analysis perspective, the compounds predicted to be the most important for anti-OA potential in PL were piperlongumine, piperlonguminine, and piperine. Based on their multi-target, multi-pathway pharmacology, they were predicted to exert their inhibitory effects on OA through six targets, primarily F2R, F3, MMP1, MMP2, MMP9, and PTGS2, and three major signaling pathways: “pathways in cancer,” “complement and coagulation cascades,” and “IL-17 signaling pathway.” Meanwhile, PL exhibited significant anti-inflammatory activity compared to the that of the control by reducing NO levels in RAW264.7 and effectively inhibited the expression of various mRNA and proteins, including F2R, F3, IL-17A, MMP-1, MMP-2, MMP-9, and PTGS2. PL showed a statistically significant effect on writhing responses *in vivo*, an apparent pathological ameliorating effect in the weight-bearing test, and amelioration of knee joint degradation in the OA-induced rats. After 24 days of treatment with PLE, a significant improvement was observed in the weight-bearing capacity of rat compared to that of the MIAs, which suggests that PLE has an analgesic effect against pain associated with OA.

PL also showed statistically significant dose-dependent effects on inflammatory cytokine levels in the serum of OA rats. These results suggest that PL may contribute to the reduction of pain and functional impairment in OA, as well as the suppression of progressive joint destruction, owing to its potent and varied anti-inflammatory activities. Cartilage loss is a predominant indicator of degenerative OA, and cartilage erosion observed in MIA rats is similar to the pathological features of human degenerative OA (Hayer et al., 2016). In addition, the experimental results of this study demonstrated marked consistency with the network analysis predictions of the multi-target, multi-pathway, and multi-compound effects of PL.

In the inflammatory pathology of OA, chondrocytes in the affected joints produce significant amounts of NO, which mediate the production of inflammatory mediators, angiogenesis, and cartilage destruction (Farrell et al., 1992; Sanchez-Lopez et al., 2022b). The synovial fluid of patients with OA contains high levels of nitrite and inducible nitric oxide synthase (iNOS), an enzyme involved in NO production. Blocking iNOS can prevent the development of OA in dogs by dramatically reducing catabolic and pro-inflammatory factors in the joints (Melchiorri et al., 1998; Pelletier et al., 1999; Sanchez-Lopez et al., 2022b). In this study, hub gene targets predicted by network analysis and experimentally investigated to support the anti-OA activity of PL were consistently associated with the inflammation-based OA pathology described above. This provided logical support for the observation that PL showed dose-dependent superiority over indomethacin in terms of anti-inflammatory activity against targets such as F2R, MMP1, and PTGS2. F2R is a thrombin receptor, also known as proteinase-activated receptor-1 (PAR1), which induces the release of prostaglandin E2 (PGE2) and phosphorylation of MAP kinases; upregulation of PTGS2 and is strongly associated with osteoblast function and repair of various bone injuries (Maeda et al., 2015; Sato et al., 2016a). Furthermore, overexpression of F2R negatively affects both osteoclast formation and regulation of function, making it a potential target for the treatment of bone diseases such as osteoporosis and suggesting its potential utility in progressive bone destruction in OA; therefore, we investigated F2R in this study (Zhang et al., 2020). Since interstitial collagenases such as MMP1 are critical in the pathogenesis of OA, therapeutic strategies to impede these targets have also been actively investigated, although it is known that modulation of these targets does not inhibit the inflammatory pathology of OA (Karila et al., 2022). Nevertheless, MMP1 has been strongly associated with pain in symptomatic knee OA in clinical studies and is involved in accelerating osteogenic differentiation, which may contribute to the direct suppression of OA-induced pain and the restoration of damaged joint tissue (Wyatt et al., 2019; Wu et al., 2020). On the other hand, PTGS2, also known as cyclooxygenase-2, is a well-known pathological factor whose increased expression in osteocytes of the subchondral bone is associated with both OA and rheumatoid arthritis (RA) (Tu et al., 2019). Therefore, PTGS2 blockade is expected to delay the course of both subchondral bone destruction and joint structural pathologies in OA, such as cartilage degeneration (Chang et al., 2021). Several PTGS2 inhibitors have already been shown to relieve pain and inflammation associated with OA; however, there is no evidence that they can halt disease progression. The results of this study, combined with recent preclinical evidence for highly selective PTGS2 inhibitors, suggest that PTGS2 may be a promising therapeutic target to inhibit both symptoms and disease progression in OA, and merits further investigation (Timur et al., 2020; Su et al., 2022). In addition, the gene targets predicted and investigated in this study, including F3, MMP2, and MMP9, consistently supported OA mitigation *in vivo experiments*. In this experiment, PLE reduced the writhing response, a quantitative indicator of peripheral pain, in AA-injected mice in a dose-dependent manner compared to the positive control (Sugita et al., 2016). By reducing the writhing response in AA-induced mice, the analgesic effect of PLE on peripheral pain observed in this study may be due to providing pain relief in MIA rats.

In this study, network analysis predicted pharmacological targets, and the positive results in rats based on these targets were also supported by the signaling pathways and multi-component information through which PL acts. In this study, we predicted that PTGS2, iNOS, and MMPs, which are key OA therapeutic targets of PL, were mainly related to cancer pathways. More specifically, among the aforementioned molecular pathways, these targets appear to be primarily involved in angiogenesis signaling. Increased angiogenesis in the subchondral bone due to vascular invasion of avascular cartilage is the most widely recognized pathological characteristic of OA (MacDonald et al., 2018). Previous studies have reported that in mice with medial meniscal instability, subchondral bone neovascularization occurs at the pre-osteoarthritic stage, before articular cartilage damage (Su et al., 2020). Thus, selective inhibition of synovial angiogenesis has emerged as an important therapeutic target for both OA and RA. In this context, PL could potentially be developed as a treatment for joint diseases that simultaneously inhibits joint inflammation and synovial angiogenesis. In contrast, PL acts on several proteinase-activated receptors during OA treatment, as confirmed by network analysis and experimental studies. KEGG enrichment analysis confirmed that these targets were predominantly involved in the regulation of platelet activation within the complement and coagulation pathways. This mechanism of action may have been observed in a previous study showing that thrombin, a cytokine encoded by the F2 gene, can modulate the mechanism of action in mouse MC3T3-E1 osteoblasts (Sato et al., 2016b). In the previous study, thrombin-stimulated osteoblasts produced a monocyte chemoattractant protein that induced the migration of macrophage RAW264 cells; this effect was inhibited by a selective non-peptide thrombin receptor inhibitor. These results indicate that thrombin may be involved in the regulation of osteoblast function, in addition to blood coagulation. Therefore, this gene may serve as a target for therapeutic interventions to inhibit osteoporotic changes in OA. This partially explains why PL may inhibit joint destruction in patients with OA. In addition, this study predicted that MMP1, MMP3, MMP9, and MMP13, which are important targets of PL, are involved in tissue remodeling signaling in the IL-17 pathway. This finding was investigated by experimental studies. These results, together with previous bioinformatic studies showing that IL-17A is highly expressed in synovitis and chondrocyte death in OA mouse models, partially support the observation in this study that PL simultaneously alleviates two OA phenotypes, synovitis, and cartilage destruction (Yang L. et al., 2023). Furthermore, these findings are consistent with previous observations that IL-17A is involved in immune, angiogenic, and complement pathways in both chondrocytes and synovial fibroblasts from patients with late-stage OA (Mimpen et al., 2021).

The major druggable compounds identified in this study could explain the multifaceted anti-OA therapeutic effects of PL, as discussed above. Piperlonguminine, which exhibits high binding affinity to hub targets, has been reported to have potent activities such as inhibition of vascular inflammation by regulating TNF-α and NF-κB production, as confirmed in this study, and anti-proliferative effects against drug-resistant cancer cells by modulating the Akt/mTOR signaling pathways (Lee et al., 2013; Zhu et al., 2020). Piperlongumine has been widely reported to have multiple mechanisms of action that support our findings, including anti-inflammatory, antiplatelet aggregation, and anti-senolytic activities (Zhu et al., 2021). Similarly, the anti-inflammatory activity of piperine, based on the inhibition of MMP-3, MMP-13, iNOS, and PTGS2 production in human OA chondrocytes, directly underpins the findings of this study (Ying et al., 2013).

The results of this study are the first report to confirm the potential value of PL as a DMOAD candidate for further research in the future. However, this possibility should not be given more than exploratory significance at this time because of the various study limitations listed below. First, the network analysis used in this study is not intended to be more than a hypothesis-generating tool. Network analysis provides rich information about the multi-component and multi-target actions of natural products, which may contribute to a more precise and efficient experimental design and therefore framing of subsequent studies. However, considerable heterogeneity exists among the databases used in this methodology, and even small variations in the network science metrics used in the analysis can lead to very different results. Therefore, from an information science perspective, it is required to simultaneously use advanced analysis techniques such as molecular docking and molecular dynamics to obtain more reproducible results in this research network analysis. Simultaneously, it is necessary to interpret the results by recognizing that the efficacy evaluation of natural products, including those obtained in this study, can only reach reliable conclusions through laboratory studies. Second, molecular docking analysis was performed to complement the results of the network analysis. However, a distinct limitation of this study is that the *in silico* approach was aimed at achieving an exploratory analysis in order to confirm the initiation value of PL in animal models of OA. Therefore, molecular dynamics simulations, which are equivalent to a more complete bioinformatic validation, were not performed. Molecular dynamics methods, such as Molecular Mechanics/Poisson-Boltzmann Surface area and molecular mechanics/generalized Born surface area, are known to perform more accurate calculations of binding free energies, and we plan to use them in our planned follow-up studies of PL, complemented by more in-depth experimental techniques. Third, this study did not provide a definitive conclusion regarding whether PL can completely inhibit the destructive skeletal pathology of OA. To be recognized as a promising DMOAD candidate, it is necessary to clearly demonstrate its effect on the inflammatory pathology of OA and the suppression of symptoms such as pain and functional disability, as well as on the progressive pathology of OA itself. Based on the promising pharmacological effects of PL on OA identified in this study, we plan to design and conduct a follow-up study in order to confirm its efficacy in inhibiting progressive cartilage destruction and osteoporotic changes. Finally, ignavine was one of the leading active components of PL predicted in this study. This compound has recently gained attention as a multiple opioid receptor modulator in various pain-related conditions. Although this may have contributed positively to the analgesia experiments in this study, HPLC analysis could not confirm its content because of difficulties in obtaining standards. Future studies should address these limitations to confirm and extend the promising results of the present study. A successful follow-up study that fully compensates for the aforementioned limitations is expected to reveal the efficacy of PL, a valuable medicinal plant with multiple indications. Additionally, it may reveal the potential of natural product-based DMOADs, which have not been successful thus far.

## 5 Conclusion

Based on the observations of the present study, we conclude that PL has broad anti-inflammatory effects that inhibit the overall pathology of OA. Network analysis predictions suggest that the treatment of OA with PL involves targets related to inflammatory mediators, angiogenesis, and joint destruction. *In vivo* and *in vitro* studies have shown that PL can attenuate pain and functional loss in OA and prevent knee joint destruction in OA rats induced by MIA injection. In addition, oral administration of PL causes broad inhibition of proinflammatory cytokines. In conclusion, PL has a significant potential for the treatment of OA and deserves further investigation as a DMOAD candidate.

## Data Availability

The data indicated in the study are deposited in the GitHub repository, accession number GU-DL0001.
