# Supplementary material for: Network analysis, in vivo, and in vitro experiments identified the mechanisms by which Piper longum L. [Piperaceae] alleviates cartilage destruction, joint inflammation, and arthritic pain
Source: Front Pharmacol. 2024 Jan 24;14:1282943. doi: 10.3389/fphar.2023.1282943 (PMC10847597; doi:10.3389/fphar.2023.1282943)
Supplement: Supplementary file 1 [file Table2.DOCX]

Network pharmacology, *in vivo* and *in vitro* experiments identified the mechanisms by which the *Piper longum* Linné alleviates cartilage destruction, joint inflammation, and arthritic pain

**Hee-Geun Jo ^1,2, †^, Chae Yun Baek ^1,†^, Donghun Lee ^1,*^, Ho-Sueb Song^3,*^**

^1^ Department of Herbal Pharmacology, College of Korean Medicine, Gachon University, 1342 Seongnamdae-ro, Sujeong-gu, Seongnam-si, 13120, Republic of Korea

^2^Naturalis Inc. 6, Daewangpangyo-ro, Bundang-gu, Seongnam-si 13549, Gyeonggi-do, Republic of Korea

^3^ Department of Acupuncture & Moxibustion Medicine, College of Korean Medicine, Gachon University 1342 Seongnamdae-ro, Sujeong-gu, Seongnam-si, Republic of Korea

†These authors contributed equally to this work and share first authorship

*** Correspondence:**

Donghun Lee
[dlee@gachon.ac.kr](mailto:dlee@gachon.ac.kr)

Ho-Sueb Song

[hssong70@gachon.ac.kr](mailto:hssong70@gachon.ac.kr)

Keywords: *Piper longum* Linné; network pharmacology; experimental verification; osteoarthritis; East Asian herbal medicine; antioxidants

**Supplementary table**

**S1, S2, S4, S5, S6, S7, S8, S9, S10, and S11**

**Supplementary Table S1**. mRNA primer sequence for OA-induced cartilage tissues

| F2R | F | GAGGATGTATGCTACGCCG |
| --- | --- | --- |
|  | R | TCGAGCGGTATGCTTTCAT |
| F3 | F | CTCCAGGGAAAGCGTTTAA |
|  | R | CCAGTGCATTTGTATTTCC |
| IL-6 | F | TCCGCAAGAGACTTCCAGC |
|  | R | CCTCCGACTTGTGAAGTGG |
| IL-17A | F | AGAATTCCATCCATGTGCCT |
|  | R | CACTGAACTTTGAGGGATGA |
| MMP-1 | F | AACTTGGGTGAAGACGTCCA |
|  | R | TCCTGTCACTTTCAGCCCAA |
| MMP-2 | F | ATGGAGGCACGATTGGTCT |
|  | R | CGCCGGGGAACTTGATGAT |
| MMP-3 | F | GTACGGCTGTGTGCTCATCC |
|  | R | TCAGCCCAAGGAACTTCTGC |
| MMP-9 | F | GCCAGCCGACTTATGTGGT |
|  | R | TGGGCTGCCCGAGTGTAAC |
| MMP-13 | F | ACCTTCTTCTTGTTGAGTTGGA |
|  | R | CTGCATTTCTCGGAGTCTA |
| NOS2 | F | AGTCAACTACAAGCCCCACG |
|  | R | GCAGCTTGTCCAGGGATTCT |
| PTGS2 | F | GTTCCAACCCATGTCAAAAC |
|  | R | TGTCAGGAATCTCGGCGTAG |
| TNF-α | F | GCATGATCCGAGATGTGGAA |
|  | R | GATGAGAGGGAGCCCATTTG |
| GAPDH | F | CTTGTGACAAAGTGGACATTGTT |
|  | R | TGACCAGCTTCCCATTCTC |
| F2R: coagulation factor II thrombin receptor, F3: coagulation factor III, IL: interleukin, MMP: matrix metalloproteinase, NOS: nitric oxide synthase, PTGS2: prostaglandin-endoperoxide synthase 2, TNF-α: tumor necrosis factor-alpha, GAPDH: glyceraldehyde 3-phosphate dehydrogenase | | |

**Supplementary Table S2**. mRNA primer sequence for LPS-stimulated RAW264.7 cells

| F2R | F | TATAAGCCTCCCTCCTCACA |
| --- | --- | --- |
|  | R | GAGGAAGGCTGACAATGAAC |
| F3 | F | ATTCCAGAGAAAGCGTTTAA |
|  | R | GTCTGTGGTCGAGAAGCACT |
| IL-1β | F | CCAGCTTCAAATCTCGCAGC |
|  | R | GTGCTCATGTCCTCATCCTGG |
| IL-6 | F | CACTTCACAAGTCGGAGGCT |
|  | R | CAAGTGCATCATCGTTGTTC |
| IL-17A | F | GAAGGTCAACCTCAAAGTCT |
|  | R | CAGAGGGATATCTATCAGGG |
| MMP-1 | F | ATGCCTAGCCTTCCTTTGCT |
|  | R | TTCCAGGTATTTCCAGACTG |
| MMP-2 | F | ATCGCCCATCATCAAGTTCC |
|  | R | AGGTTGCAACTCTCCTTGG |
| MMP-3 | F | AAGTTCCTCGGGTTGGAGAT |
|  | R | ACCAACATCAGGAACACCAC |
| MMP-9 | F | CAATCAATTCCGGTCTTCGA |
|  | R | GGTTAGCAAGAAATCACCAGA |
| MMP-13 | F | AACCAAGATGTGGAGTGCCT |
|  | R | GACCAGACCTTGAAGGCTTT |
| NOS2 | F | ACCAAGATGGCCTGGAGGAA |
|  | R | CCGACCTGATGTTGCCATTG |
| PTGS2 | F | ATCCATGTCAAAACCGTGGG |
|  | R | TTGGGGTGGGCTTCAGCAG |
| PGE2 | F | CTGGTAACGGAATTGGTGC |
|  | R | TGGCCAGACTAAAGAAGGTC |
| TNF-α | F | GAGAAGTTCCCAAATGGCCT |
|  | R | AGCCACTCCAGCTGCTCCT |
| GAPDH | F | ATGGTGAAGGTCGGTGTG |
|  | R | GCCGTGAGTGGAGTCATAC |
| F2R: coagulation factor II thrombin receptor, F3: coagulation factor III, IL: interleukin, MMP: matrix metalloproteinase, NOS: nitric oxide synthase, PTGS2: prostaglandin-endoperoxide synthase 2, PGE2: prostaglandin E2, TNF-α: tumor necrosis factor-alpha, GAPDH: glyceraldehyde 3-  phosphate dehydrogenase. | | |

**Supplementary Table S4**. Macroscopic score of cartilage erosion

| Grade | Cartilage appearance |
| --- | --- |
| 0 | Normal appearance in cartilage surface |
| 1 | Slight yellowish discoloration of the surface or slight fibrillation |
| 2 | Erosion reaching the superficial or middle layers of the cartilage |
| 3 | Extensive erosions reaching down to the subchondral bone |
| 4 | Massive erosions with extensive exposure of subchondral bone |

**Supplementary Table S5.** Weight bearing index (WBI)

|  | **NT** | **MIA** | **INDO 3** | **PLE 100** | **PLE 300** |
| --- | --- | --- | --- | --- | --- |
| Day 0 | 51.15±0.46 | 50.04±0.40 | 49.62±0.53 | 49.66±0.35 | 50.09±0.53 |
| Day 3 | 50.94±0.09### | 27.92±1.79 | 30.62±1.61 | 29.27±1.73 | 28.08±1.64 |
| Day 7 | 50.45±0.29### | 20.51±1.93 | 37.86±2.36*** | 28.20±2.70 | 28.06±1.84 |
| Day 10 | 50.46±0.07### | 26.03±2.72 | 41.64±1.99*** | 35.45±2.60* | 36.72±2.08** |
| Day 14 | 49.97±0.76### | 22.37±1.39 | 36.79±1.24*** | 27.33±1.93 | 34.91±1.72*** |

All values are expressed as Mean±SD; * p <0.05, ** p <0.01, *** p <0.001 compared to MIA; p-value obtained by two-way ANOVA followed by Tukey’s multiple comparison test.

|  | **Independent Variable** | **Source of Variation** |
| --- | --- | --- |
| 2-way ANOVA | Time | F (3.709, 40.80) = 37.15, P=0.001 |
|  | Treatment | F (2.147, 23.62) = 109.0, P=0.001 |
|  | Time/Treatment | F (5.292, 29.10) = 5.048, P=0.002 |

**Supplementary Table S6.** Macroscopic score

| **Groups** | **Macroscopic score** |
| --- | --- |
| NT | - |
| MIA | 3.67±0.70 |
| INDO 3 | 1.67±0.71^***^ |
| PLE 100 | 2.67±0.71^*^ |
| PLE 300 | 1.67±0.71^***^ |

All values are expressed as Mean±SD; *** p <0.001 compared to control; p-value obtained by one-way ANOVA followed by Dunnett's post hoc test.

|  | **Independent Variable** | **Source of Variation** |
| --- | --- | --- |
| 1-way ANOVA | sample | F (3, 32) = 13.07, P<0.0001 |

**Supplementary Table S7.** Writhing test response

| **Groups** | **Writhing responses** |
| --- | --- |
| control | 100±15.89 |
| IBU 200 | 44.17±19.66^***^ |
| PLE 200 | 35.75±26.24^***^ |
| PLE 600 | 32.14±20.78^***^ |

All values are expressed as Mean±SD; *** p <0.001 compared to control; p-value obtained by one-way ANOVA followed by Dunnett's post hoc test.

|  | **Independent Variable** | **Source of Variation** |
| --- | --- | --- |
| 1-way ANOVA | Sample | F (3, 27) = 18.50, P<0.001 |

**Supplementary Table S8.** Cell Viability and Nitric Oxide

| **Groups** | **Cell Viability** | **Nitric Oxide** |
| --- | --- | --- |
| NT | - | 11.26±0.36 |
| Control | 100±5.2 | 100±1.73^###^ |
| DEX 1 | 91.09±0.98 | 95.71±1.14^*^ |
| PLE 10 | 104.1±0.97 | 101.7±0.71 |
| PLE30 | 106.8±6.37 | 97.43±1.83 |
| PLE 100 | 112.8±6.05 | 91.89±6.05^***^ |
| PLE 300 | 116.7±8.31^*^ | 88.69±1.56^***^ |

All values are expressed as Mean±SD; *** p <0.001 compared to control; p-value obtained by one-way ANOVA followed by Dunnett's post hoc test.

|  | **Independent Variable** | **Source of Variation** |
| --- | --- | --- |
| 1-way ANOVA | Cell Viability | F (5, 12) = 8.486, p=0.0012 |
|  | Nitric Oxide | F (5, 12) = 33.53, P<0.0001 |

**Supplementary Table S9.** Serum analysis

| **Groups** | **TNF-α** | **IL-6** |
| --- | --- | --- |
| NT | 0.0017±0.00 | 0.0002±0.00 |
| MIA | 100±8.228^###^ | 100±15.43^###^ |
| INDO 3 | 22.38±14.95^***^ | 31.48±5.47*** |
| PLE 100 | 30.27±12.97^***^ | 47.74±3.29*** |
| PLE 300 | 15.92±12.29^***^ | 32.17±6.05*** |

All values are expressed as Mean±SD; *** p <0.001 compared to control; p-value obtained by one-way ANOVA followed by Dunnett's post hoc test.

|  | **Independent Variable** | **Source of Variation** |
| --- | --- | --- |
| 1-way ANOVA | TNF-α | F (4, 37) = 105.1, P<0.001 |
|  | IL-6 | F (3, 35) = 123.8, P<0.001 |

**Supplementary Table S10.** qRT-PCR analysis of articular cartilage induced rats

| **Groups** | | **F2R** | | | **F3** | | | **IL-6** | | | **IL-17A** | | |
| --- | --- | --- | --- | --- | --- | --- | --- | --- | --- | --- | --- | --- | --- |
| NT | | 0.002±0.00 | | | 0.002±0.00 | | | 0.002±0.00 | | | 0.001±0.00 | | |
| MIA | | 1.00±0.41^###^ | | | 1.00±0.21^###^ | | | 1.00±0.22^###^ | | | 1.00±0.30^###^ | | |
| INDO 3 | | 0.047±0.07^***^ | | | 0.350±0.21^***^ | | | 0.352±0.28^***^ | | | 0.018±0.02^***^ | | |
| PLE 100 | | 0.085±0.14^***^ | | | 0.508±0.29^***^ | | | 0.468±0.25^***^ | | | 0.040±0.03^***^ | | |
| PLE 300 | | 0.001±0.00^***^ | | | 0.160±0.08^***^ | | | 0.433±0.18^***^ | | | 0.015±0.01^***^ | | |
| **Groups** | **MMP1** | | | **MMP2** | | | **MMP3** | | | **MMP9** | | |  |
| NT | 0.002±0.00 | | | 0.002±0.00 | | | 0.002±0.00 | | | 0.002±0.00 | | |  |
| MIA | 1.00±0.36^###^ | | | 1.00±0.48^###^ | | | 1.00±0.33^###^ | | | 1.00±0.17^###^ | | |  |
| INDO 3 | 0.303±0.17^***^ | | | 0.280±0.22^***^ | | | 0.425±0.34^***^ | | | 0.115±0.16^***^ | | |  |
| PLE 100 | 0.381±0.25^***^ | | | 0.864±0.13* | | | 0.685±0.34^*^ | | | 0.0307±0.04^***^ | | |  |
| PLE 300 | 0.267±0.11^***^ | | | 0.265±0.27^***^ | | | 0.442±0.18^***^ | | | 0.020±0.03^***^ | | |  |
| **Groups** | **MMP13** | | **NOS2** | | | **PTGS2** | | | **TNF-α** | | |  |  |
| NT | 0.001±0.00 | | 0.001±0.00 | | | 0.000±0.00 | | | 0.002±0.00 | | |  |  |
| MIA | 1.00±0.30^###^ | | 1.00±0.53^###^ | | | 1.00±0.18^###^ | | | 1.00±0.42^###^ | | |  |  |
| INDO 3 | 0.206±0.11^***^ | | 0.356±0.26^***^ | | | 0.108±0.15^***^ | | | 0.279±0.31^***^ | | |  |  |
| PLE 100 | 0.538±0.27^***^ | | 0.197±0.19^***^ | | | 0.192±0.38^***^ | | | 0.360±0.22^***^ | | |  |  |
| PLE 300 | 0.100±0.10^***^ | | 0.129±0.13^***^ | | | 0.004±0.01^***^ | | | 0.143±0.09^***^ | | |  |  |

All values are expressed as Mean±SD; *** p <0.001 compared to control; p-value obtained by one-way ANOVA followed by Dunnett's post hoc test.

|  | **Independent Variable** | **Source of Variation** |
| --- | --- | --- |
| 1-way ANOVA | F2R | F (3, 32) = 41.62, P<0.0001 |
|  | F3 | F (3, 32) = 28.93, P<0.0001 |
|  | IL-6 | F (3, 29) = 13.55, P<0.0001 |
|  | IL-17A | F (3, 29) = 82.33, P<0.0001 |
|  | MMP1 | F (3, 32) = 18.53, P<0.0001 |
|  | MMP2 | F (3, 32) = 14.53, P<0.0001 |
|  | MMP3 | F (3, 29) = 6.322, P=0.002 |
|  | MMP9 | F (3, 32) = 137.8, P<0.0001 |
|  | MMP13 | F (3, 32) = 31.71, P<0.0001 |
|  | NOS2 | F (3, 32) = 14.47, P<0.0001 |
|  | PTGS2 | F (3, 32) = 37.53, P<0.0001 |
|  | TNF-α | F (3, 29) = 14.45, P<0.0001 |

**Supplementary Table S11.** qRT-PCR analysis of LPS-induced RAW264.7 cell

| **Groups** | | **F2R** | | | **F3** | | | **IL-1β** | | | **IL-6** | | | **Il-17A** |
| --- | --- | --- | --- | --- | --- | --- | --- | --- | --- | --- | --- | --- | --- | --- |
| NT | | 0.003±0.00 | | | 0.003±0.00 | | | 0.003±0.00 | | | 0.0002±0.00 | | | 0.003±0.00 |
| Control | | 1.00±0.52^###^ | | | 1.00±0.05^###^ | | | 1.00±0.14^###^ | | | 1.00±0.02^###^ | | | 1.00±0.11^###^ |
| DEX 1 | | 0.159±0.16^***^ | | | 0.352±0.02^***^ | | | 0.619±0.28^***^ | | | 0.629±0.05^***^ | | | 0.262±0.01^***^ |
| PLE 30 | | 0.171±0.13^***^ | | | 0.757±0.18^***^ | | | 0.677±0.23^*^ | | | 0.100±0.04 | | | 0.552±0.01^***^ |
| PLE 100 | | 0.109±0.04^***^ | | | 0.563±0.04^***^ | | | 0.388±0.08^***^ | | | 0.732±0.05^***^ | | | 0.240±0.09^***^ |
| PLE 300 | | 0.0308±0.04^***^ | | | 0.282±0.10^***^ | | | 0.308±0.16^***^ | | | 0.596±0.04^***^ | | | 0.163±0.08^***^ |
| **Groups** | **MMP1** | | | **MMP2** | | | **MMP3** | | | **MMP9** | | | **MMP13** | |
| NT | 0.003±0.00 | | | 0.000±0.00 | | | 0.003±0.00 | | | 0.000±0.00 | | | 0.022±0.00 | |
| Control | 1.00±0.07^###^ | | | 1.00±0.08^###^ | | | 1.00±0.05^###^ | | | 1.00±0.14^###^ | | | 1.00±0.10^###^ | |
| DEX 1 | 0.453±0.22^***^ | | | 0.895±0.10^***^ | | | 0.325±0.09^***^ | | | 0.737±0.01^***^ | | | 0.614±0.10^***^ | |
| PLE 30 | 0.620±0.11^***^ | | | 0.615±0.04^***^ | | | 0.528±0.04^***^ | | | 0.866±0.07^**^ | | | 0.593±0.13^***^ | |
| PLE 100 | 0.377±0.04^***^ | | | 0.553±0.07^***^ | | | 0.395±0.02^***^ | | | 0.779±0.10^***^ | | | 0.545±0.03^***^ | |
| PLE 300 | 0.253±0.03^***^ | | | 0.410±0.00^***^ | | | 0.156±0.02^***^ | | | 0.629±0.01^***^ | | | 0.144±0.02^***^ | |
| **Groups** | | | **NOS2** | | | **PTGS2** | | | **PGE2** | | | **TNF-α** | | |
| NT | | | 0.003±0.00 | | | 0.000±0.00 | | | 0.003±0.00 | | | 0.010±0.00 | | |
| Control | | | 1.00±0.08^###^ | | | 1.00±0.04^###^ | | | 1.00±0.14^###^ | | | 1.00±0.21^###^ | | |
| DEX 1 | | | 0.773±0.23^**^ | | | 0.325±0.09^***^ | | | 0.673±0.07^***^ | | | 0.629±0.01^***^ | | |
| PLE 30 | | | 0.961±0.17 | | | 0.758±0.02^***^ | | | 0.633±0.07^***^ | | | 0.817±0.13^*^ | | |
| PLE 100 | | | 0.773±0.23^**^ | | | 0.652±0.07^***^ | | | 0.431±0.01^***^ | | | 0.702±0.23^***^ | | |
| PLE 300 | | | 0.399±0.08^***^ | | | 0.167±0.01^***^ | | | 0.251±0.03^***^ | | | 0.239±0.11^***^ | | |

All values are expressed as Mean±SD; *** p <0.001 compared to control; p-value obtained by one-way ANOVA followed by Dunnett's post hoc test.

|  | **Independent Variable** | **Source of Variation** |
| --- | --- | --- |
| 1-way ANOVA | F2R | F (4, 10) = 7.564, P=0.005 |
|  | F3 | F (4, 10) = 27.16, P<0.001 |
|  | Il-1β | F (4, 10) = 5.997, P=0.01 |
|  | IL-6 | F (4, 10) = 60.90, P<0.001 |
|  | IL-17A | F (4, 10) = 68.99, P<0.001 |
|  | MMP1 | F (4, 10) = 18.67, P<0.001 |
|  | MMP2 | F (4, 10) = 38.29, P<0.001 |
|  | MMP3 | F (4, 10) = 109.2, P<0.001 |
|  | MMP9 | F (4, 10) = 8.372, P=0.003 |
|  | MMP13 | F (4, 10) = 47.54, P<0.001 |
|  | NOS2 | F (4, 10) = 5.895, P=0.01 |
|  | PTGS2 | F (4, 10) = 105.2, P<0.001 |
|  | PGE2 | F (4, 10) = 37.19, P<0.001 |
|  | TNF-α | F (4, 10) = 9.716, P=0.0018 |
